# Silencing CTNND1 Mediates Triple-Negative Breast Cancer Bone Metastasis via Upregulating CXCR4/CXCL12 Axis and Neutrophils Infiltration in Bone

**DOI:** 10.3390/cancers13225703

**Published:** 2021-11-17

**Authors:** Qun Lin, Xiaolin Fang, Gehao Liang, Qing Luo, Yinghuan Cen, Yu Shi, Shijie Jia, Juanmei Li, Wenqian Yang, Andrew J. Sanders, Chang Gong, Wenguo Jiang

**Affiliations:** 1 Breast Tumor Center, Sun Yat-sen Memorial Hospital, Sun Yat-sen University, Guangzhou 510120, China; linq27@mail2.sysu.edu.cn (Q.L.); fangxlin3@mail.sysu.edu.cn (X.F.); lianggeh@mail2.sysu.edu.cn (G.L.); luoq63@mail2.sysu.edu.cn (Q.L.); cenyh@mail2.sysu.edu.cn (Y.C.); shiy77@mail2.sysu.edu.cn (Y.S.); jiashj3@mail2.sysu.edu.cn (S.J.); lijm95@mail.sysu.edu.cn (J.L.); yangwq29@mail.sysu.edu.cn (W.Y.); 2 Guangdong Provincial Key Laboratory of Malignant Tumor Epigenetics and Gene Regulation, Sun Yat-sen Memorial Hospital, Sun Yat-sen University, Guangzhou 510120, China; 3 Department of Breast Oncology, Sun Yat-sen University Cancer Center, Sun Yat-sen University, Guangzhou 510080, China; 4 Cardiff China Medical Research Collaborative, Cardiff University School of Medicine, Cardiff University, Heath Park, Cardiff CF14 4XN, UK; sandersaj1@cardiff.ac.uk

**Keywords:** CTNND1, bone metastasis, epithelial–mesenchymal transformation, CXCR4, neutrophils

## Abstract

**Simple Summary:**

Distant metastasis, especially bone metastasis, is the major cause of death in breast cancer patients. However, breast cancer patients with bone metastasis are frequently complicated by delayed intervention as clinically bone metastasis cannot be detected early enough. Here, we report that CTNND1 is downregulated in both primary tumors and metastatic bone lesions of patients with triple-negative breast cancer (TNBC). Decreased CTNND1 is a crucial intrinsic contributor to homing to the bones and the survival of the breast cancer cells in the bone microenvironment. Thus, CTNND1 may be a novel biomarker for early predicting bone metastasis of triple-negative breast cancer.

**Abstract:**

Bone metastasis from triple-negative breast cancer (TNBC) frequently results in poorer prognosis than other types of breast cancer due to the delay in diagnosis and intervention, lack of effective treatments and more skeletal-related complications. In the present study, we identified CTNND1 as a most reduced molecule in metastatic bone lesion from TNBC by way of high throughput sequencing of TNBC samples. In vivo experiments revealed that knockdown of CTNND1 enhanced tumor cells metastasis to bones and also increased neutrophils infiltration in bones. In vitro, we demonstrated that knockdown of CTNND1 accelerated epithelial–mesenchymal transformation (EMT) of tumor cells and their recruitment to bones. The involvement by CTNND1 in EMT and bone homing was achieved by upregulating CXCR4 via activating the PI3K/AKT/HIF-1αpathway. Moreover, TNBC cells with reduced expression of CTNND1 elicited cytotoxic T-cells responses through accelerating neutrophils infiltration by secreting more GM-CSF and IL-8. Clinically, patients with triple-negative breast cancer and lower level of CTNND1 had shorter overall survival (OS) and distant metastasis-free survival (DMFS). It was concluded that downregulation of CTNND1 played a critical role in facilitating bone metastasis of TNBC and that CTNND1 might be a potential biomarker for predicting the risk of bone metastases in TNBC.

## 1. Introduction

Female breast cancer (BC) is a heterogenous and complex disease and has become the leading cancer type in global cancer incidence [1]. In BC patients, distant metastases, rather than the primary tumors, are the main cause of death. Of the location of distant metastases, bone is the most common site [2]. Clinically, current treatments for bone metastasis of breast cancer involve multiple and often combined approaches ranging from conventional surgical, drug and radiation therapies to newly developed biological drugs, targeted treatment and gene therapy [3,4,5]. Amongst the subtypes of breast cancer, triple-negative breast cancer (TNBC) is a recognized subtype that is difficult to treat, as those treatments targeting these receptors are not effective for this tumor type [5,6]. In addition, compared with the luminal subtype, TNBC patients with bone metastasis have poorer outcomes as the risk of bone metastasis cannot be early and accurately predicted [3,7,8,9]. Thus, new predictive biomarkers and special treatment approaches are urgently needed to be identified in TNBC patients with high risk of bone metastasis.

P120 catenin (p120^ctn^), the protein encoded by Catenin Delta 1 (CTNND1), is a member of a subfamily of armadillo (ARM) repeating containing proteins. The p120^ctn^ protein is best known for its interaction with E-cadherin, leading to formation of the adherens junctions (AJ) through the juxtamembrane domain (JMD), which is essential for cadherin stability [10]. Binding of p120^ctn^ and E-cadherin mediates cell–cell adhesion and its dysregulation results in disassembly of adherens junctions. Disruption of AJ is known to affect nuclear signaling pathways such as receptor tyrosine, PI3K/AKT, Rho GTPase, and HIPPO. In addition, p120^ctn^ also participates in several aspects of cell signaling, transcriptional regulation and cytoskeletal organization in various biological processes [11,12]. For example, conventional p120^ctn^ knockout (KO) in mice is embryonically lethal, and the conditional p120^ctn^ KO animals show that p120^ctn^ loss leads to inflammation of multiple organ systems [13,14,15]. Moreover, p120^ctn^ is recognized as a key factor in cancer progression by functioning as either an oncogene or a tumor suppressor, largely depending on cell types and cancer types [12]. Cytosolic translocation of p120^ctn^ due to the inactivation of E-cadherin associates with the tumor malignancy in breast, colon, lung, bladder, pancreas, etc. [16,17,18,19,20,21].

Bone metastasis is a multi-step process, and the underlying molecular mechanisms are variable and complex. The principal process of bone metastasis includes escape of cancer cells from the primary tumor, intravasation from the primary tumor site, dissemination through circulation, extravasation into the bone, and regrowth in the bone [22]. Moreover, in order to form a metastasis focus, cancer cells have to adapt to the local bone microenvironment, which consists of different cellular and matrix components including immunocompetent cells in bone [23]. In recent years, tumor cells have been found to form pre-metastatic niches in bone before metastasis by secreting factors such as interleukin-6 (IL-6), parathyroid hormone-related protein (PTHrP), and matrix metalloproteinases (MMPs). These factors would allow cancer cells to better adapt to the microenvironment of the bone [8]. In the process of homing, tumor cells overexpressing CXCR4 or CXCR7 have higher potential to survive the circulation stage, guarded by CXCL12 secreted by bone stromal cells [24,25]. After seeding and forming micrometastases in the bone, tumor cells may die, or turn into dormancy or grow to a metastatic lesion in bone. To survive and establish metastases in the bone, tumor cells may also secrete such factors as lectin galactoside-binding soluble 3 (LGALS3) in hepatocellular carcinoma (HCC) to form a “vicious cycle” which facilitates seeding and expansion of metastatic tumor cells in the bone [26]. So far, there remain a great deal of unknown mechanisms underlying the bone metastasis of breast cancer, examples including the bone metastasis characteristics of different types breast cancer with high potential of bone metastasis, the regulation of homing receptors like CXCR4 and CXCR7, and impact of the immune microenvironment in bone metastases.

In the present study, through high-throughput sequencing, we identified CTNND1 as the most downregulated molecule in TNBC patients with bone metastasis. We further demonstrated that knockdown of CTNND1 not only enhanced intrinsic metastatic functions including migration and invasion, but also facilitated tumor recruitment to bones, an event through the CXCR4/CXCL12 axis by activating PI3K/AKT/HIF-1α pathway. Moreover, the study also revealed the potential role of CTNND1 in immune microenvironment of bone, especially in neutrophil infiltration in TNBC. Our results presented CTNND1 as a potential biomarker for early predicting of the risk of bone metastasis of TNBC patients.

## 2. Materials and Methods

### 2.1. Patient Samples and Database

In our study, three independent cohorts of breast cancer patients were enrolled at Sun Yat-Sen Memorial Hospital (SYSMH). Paraffin-embedded normal tissues, tissues of primary tumors and bone metastatic tissues from twenty patients were used for IHC staining. Survival data of a TCGA TNBC cohort was downloaded from the TCGA Pan Cancer Clinical Data Resource (accessed on 14 July 2021). Survival analysis was performed by website tools (Kaplan–Meier plotter, http://kmplot.com/analysis/, accessed on 14 July 2021). Overall survival (OS) was defined as the interval between the date of diagnosis and death due to TNBC. Distant metastasis-free survival (DMFS) was defined as the time elapsed from the date when the patients underwent surgery to the date when comprehensive imaging analyses first confirmed the presence of distant metastasis.

### 2.2. Immunohistochemistry (IHC)

IHC was performed by using a SP Rabbit and Mouse HRP Kit (cw2069, Cwbio, Beijing, China), according to the manufacturer’s protocol. Primary antibodies against CTNND1(bs-7000R, 1:100, Bioss, Beijing, China), CXCR4(bs-1011R, 1:100, Bioss, Beijing, China), AKT (bsm-33325M, 1:100, Bioss, Beijing, China), p-AKT(bsm-33281M, 1:100, Bioss, Beijing, China) were used. Staining scores were determined based on both the intensity and proportion in the whole section. The staining intensity was graded according to the following standard: 1, no staining; 2, weak staining (light yellow); 3, moderate staining (yellow brown); 4, strong staining (brown).

### 2.3. RNA Isolation and qRT-PCR

TRIzol reagent (Invitrogen, Carlsbad, CA, USA) was used for collecting the total RNA of fresh-frozen breast cancer tissues or breast cancer cell lines. We subsequently used RT Super Mix (Vazyme Biotech, Nanjing, China) to reverse transcribe the RNA to cDNA, following the manufacturer’s protocol. SYBR qRT-PCR Master Mix (Vazyme Biotech, Nanjing, China) was used in the real-time quantitative polymerase chain reaction (qRT-PCR) on Light Cycler 480 II system (Roche, Basel, Switzerland). The sequence of primers in this study is shown in Table A1. The expression level of all RNA was analyzed by using β-actin as a housekeeping control.

### 2.4. RNAseq

Magzol Reagent (Magen, Guangzhou, China) was used to isolate total RNA from cells/tissues, according to the manufacturer’s protocol. The quantity and integrity of RNA yield was assessed by using the K5500 (Beijing Kaiao, China) and Agilent 2200 Tape Station (Agilent Technologies, Santa Clara, CA, USA) separately. The mRNA was enriched by oligo dT via instructions of the NEB Next^®^ Poly(A) mRNA Magnetic Isolation Module (NEB, Ipswich, MA, USA) and fragmented to approximately 200 bp. Next, the RNA fragments were subjected to first-strand and second-strand cDNA synthesis, followed by adaptor ligation and enrichment with a low cycle, according to the manufacturer’s instructions (NEB Next^®^ Ultra™ RNA Library Prep Kit for Il-lumina). The purified library products were evaluated using the Agilent 2200 Tape Station and Qubit (Thermo Fisher Scientific, Carlsbad, CA, USA). The libraries were sequenced by Illumina (Illumina, San Diego, CA, USA) with paired-end 150 bp at Ribobio Co. Ltd. (Ri-bobio, Guangzhou, China).

### 2.5. In Vivo Bone Metastasis Models

Six-to-eight-week-old C57BL/6 mice, purchased from the Guangdong medical laboratory animal center, were raised in the SPF animal facility in the Laboratory Animal Resource Center at Sun Yat-Sen University. Luciferase-labeled EO771 cells transduced with sh-CTNND1 (designated here as EO771^CTNND1KD^) or sh-control (designated as EO771^sh-controlD^) (5 × 10^5^ cells in 100 μL PBS) were injected into the left cardiac ventricle. Bone metastases were detected and quantified weekly after injection by BLI using IVIS Lumina imaging (Xenogen IVIS Lumina System, Caliper Life Sciences, Waltham, MA, USA). Mice were i.v.-injected with 300 mg per kg D-luciferin and anesthetized with 3% isoflurane ten minutes before imaging.

### 2.6. μCT Analyses

μCT (Bruker, Billeraca, MA, USA) was used for detecting bone destruction. Bones of the mice were fixed in 4% paraformaldehyde and we scanned the bones at a pixel size of 18 μm and analyzed the results according to the manufacturer’s instruction. BMD (bone mineral density) was analyzed using CTAn software (Bruker μCT). Living Image software version 3.0 (Caliper Life Sciences, Waltham, MA, USA) was used for image analysis.

### 2.7. FACS Analysis of Immune Cells in Bone Metastasis

Mice were first anaesthetized and then sacrificed by cervical dislocation. Bone marrow cells were obtained by flushing the marrow cavities with FACS buffer (PBS with 3% FBS) using an insulin syringe with a 28G needle and then passing it through a 70 μm filter to remove the bone fragments. Red blood cells were lysed by 5 mL RBC lysis buffer (Solarbio, Beijing, China). Then, cells were resuspended in FACS buffer, followed by incubation with Fc block buffer at room temperature for 15 min. Cells were suspended in 100 μL of FACS buffer and stained for the designated cell type. Cells were stained with the following antibodies at a dilution ration of 1:20:anti-CD45 APC-Cy7 (557659, Becton, Dickinson and Company, New York, NY, USA), anti-CD3e FITC (553061, Becton, Dickinson and Company, New York, NY, USA), anti-NK1.1BV650 (564143, Becton, Dickinson and Company, New York, NY, USA), anti-CD11b PE (557397, Becton, Dickinson and Company, New York, NY, USA), anti-F4/80 BV421 (565411, Becton, Dickinson and Company, New York, NY, USA), anti-CD11c Percp-Cy5.5 (560584, Becton, Dickinson and Company, New York, NY, USA), anti-Gr-1, PE-Cy7 (565033, Becton, Dickinson and Company, New York, NY, USA), anti-Ly6C APC (560595, Becton, Dickinson and Company, New York, NY, USA), anti-Ly6G Percp-Cy5.5 (560602, Becton, Dickinson and Company, New York, NY, USA), anti-CD8 Percp-Cy5.5 (Thermo, 45-0081-80, Carlsbad, CA, USA), anti-IFN-γ APC (ab275700, ABCAM, Cambridge, UK), anti-GranzymeB FITC (Thermo, 180734, Carlsbad, CA, USA), anti-Perforin PE (Thermo, 12939280, Carlsbad, CA, USA). All other reagents were from BioLegend (San Diego, CA, USA), unless indicated otherwise. The data acquired on cells were detected by multicolor flow cytometry (Gallios, Beckman Coulter or Attune NxT, Thermo, Carlsbad, CA, USA) and analyzed using FlowJo software (BD, East Rutherford, NJ, USA).

### 2.8. Neutrophils and CD8^+^ T Cells Isolation

To isolate neutrophils from bone marrow, bone marrow cells from mice were harvested in sterile Hank’s buffered salt solution (HBSS) without Ca^2+^/Mg^2+^ (14185052, Invitrogen). Red blood cells were lysed by the addition of 5 mL RBC lysis buffer (Solarbio, Beijing, China). Then, neutrophils and CD8^+^T cells were collected and purified using a Myeloid-Derived Suppressor Cell Isolation Kit, mouse (MiltenyiBiotec, 130094538, Cologne, Germany) and CD8 (TIL) MicroBeads, mouse (MiltenyiBiotec, 130116478, Cologne, Germany).

### 2.9. ChIP Assay

MDA-MB-231 cells transduced with sh-CTNND1 or sh-control were collected for ChIP assay. Cells were crosslinked in 1% formaldehyde solution for 10 min at room temperature and then added into 1 mL 1 × glycine buffer for 5 min. ChIP assay was performed by using Enzymatic Chromatin IP Kit (Cell Signaling Technology, 9003, Danvers, MA, USA), according to the manufacturer’s instructions. By using the Micrococcal Nuclease in the kit, the nucleoprotein complexes were digested to yield DNA fragments ranging from 200 to 500 bp. Two micrograms of normal IgG were used as the negative control, and anti-HIF-1α (Proteintech, 209601AP, Wuhan, China) antibodies were used for each immunoprecipitation. The immunoprecipitate was eluted and reverse-crosslinked, after which the DNA fragments were purified. Immunoprecipitated and input DNAs were subjected to qRT–PCR analysis. The primers used for amplifying the promoter of CXCR4 were forward: GAGTGCAGTCTGGGCAATCC and reverse: CGGGCGTCTTCCACGATTTTG; β-actin:forward:AGGAATGGGTGGGAAGTCAG and reverse: GGGCCAAGGACTCTTACTGT. Luciferase reporter assays were performed according to the manufacturer’s instructions (DD120501, Vazyme Biotech, Nanjing, China). Firefly and Renilla luciferase activities were detected by the Dual-Luciferase Reporter Assay System.

### 2.10. Cytokine Antibody Array

By using human Cytokine Antibody Array (R&D, ARY005B) according to the manufacturer’s instructions, cytokines were detected in media of MDA-MB-231 transduced with sh-CTNND1 or sh-control. Membranes were blocked with the blocking buffer for 45 min, and then incubated with 1 mL of samples containing protease inhibitor cocktail overnight at 4 °C. After biotin-conjugated antibody and HRP-streptavidin incubation, chemiluminescence detection was performed.

### 2.11. Generation of siRNAs and Plasmid Constructs

Sh-RNA of CTNND1 and mut-CTNND1 vectors were generated in gene 1, guangzhou, China. Sh-RNA1 target sequence: GCCACTATGAAGATGGTTA; Sh-RNA2 target sequence:GGCACCTAGTAGACAGGAT. Sh-RNA3 target sequence: GCAAGGCAGTCCATGTCAT; Sh-RNA4 target sequence: CCGACTTCATCTTTGCCAA; For mut-CTNND1 vector, we created siRNA-resistant CTNND1 cDNA by introducing silent mutations in the si-RNA target sequence of CTNND1 cDNA.

### 2.12. Cell Culture and Treatment

In the current study, we used normal human mammary epithelial cell line (MCF-10A), TNBC cell lines (MDA-MB-1833, MDA-MB-231, MDA-MB-468, BT549, Hs578T, MDA-MB-453, HCC1937), other types of breast cancer cell lines (MCF-7, T47D, BT474, SKBR3) and mouse TNBC cell line (EO771). All cell lines were obtained from the American Type Culture Collection (Manassas, VA, USA) and grown according to standard protocols.

### 2.13. Cell Migration and Invasion Assay

For cellular migration assay, 5 × 10^5^ cells in 100 μL serum-free medium were seeded in the upper chamber (Falcon, 8 μm pore size). For cells invasion assay, cells were seeded in Matrigel (BD biosciences, East Rutherford, NJ, USA)-coated 24-well tissue culture plates (Falcon, 8 μm pore size). After incubation for 8 h, the cells that had migrated to the bottom chamber were fixed with 4% formaldehyde and stained with 1% crystal violet. Then, the migrated cells in each group were calculated by using Image J software.

### 2.14. Plate Colony Formation Assay

The MDA-MB-231, MDA-MB-468 and BT549 cells, after being transduced with sh-CTNND1 or sh-control, were cultured for 3 weeks in 6-well plates (2 × 10^3^). Formaldehyde (4%) was used for fixing the colonies for 15 min, and 1% crystal violet was used to stain for 30 min. Then, calculating the colonies in each group was carried out by using Image J software.

### 2.15. Cell Counting Kit-8 Assay

MDA-MB-231, MDA-MB-468 and BT549 cells (1.5 × 10^3^), after being transduced with sh-CTNND1 or sh-control, were seeded in 96-well plates. Cellular viability of each group was determined daily for five days by a Cell Counting Kit-8 (Dojindo, Kyushu Island, Japan).

### 2.16. Western Blot Analysis

RIPA lysis buffer (BCA) kit (Lot 30342, Cwbio, Beijing, China) was used to lyse cells, and the concentration of protein was subsequently quantified. Ten percent SDS-PAGE was used in the electrophoresis process and then the protein in the gels was transferred to the PVDF membrane. After incubating with 5% non-fat milk at room temperature, primary antibody anti-GAPDH (Cell Signaling Technology, 1:1000, Danvers, MA, USA), anti-CTNND1 (Cell Signaling Technology, 1:1000, Danvers, MA, USA), anti-CXCR4 (ABCAM, 1:1000, Cambridge, UK), anti-AKT (Cell Signaling Technology, 1:1000, Danvers, MA, USA), anti-p-AKT (Cell Signaling Technology, 1:1000, Danvers, MA, USA) were respectively added to the membrane. After being incubated at 4 °C overnight, secondary antibody (Cell Signaling Technology, 1:3000, Danvers, MA, USA) was added and incubated at room temperature for 2 h. Finally, the blots were detected by an enhanced chemiluminescence kit (P90719, Millipore, MA, USA) under Image Lab Software.

### 2.17. Statistical Analysis

The experiment data were analyzed by using Student’s *t*-test or one-way ANOVA in GraphPad Prism 8 software. *p* < 0.05 was considered statistically significant.

## 3. Results

### 3.1. Reduction of CTNND1 In-Bone-Metastatic Tumors of TNBC Patients

**Figure 1:**
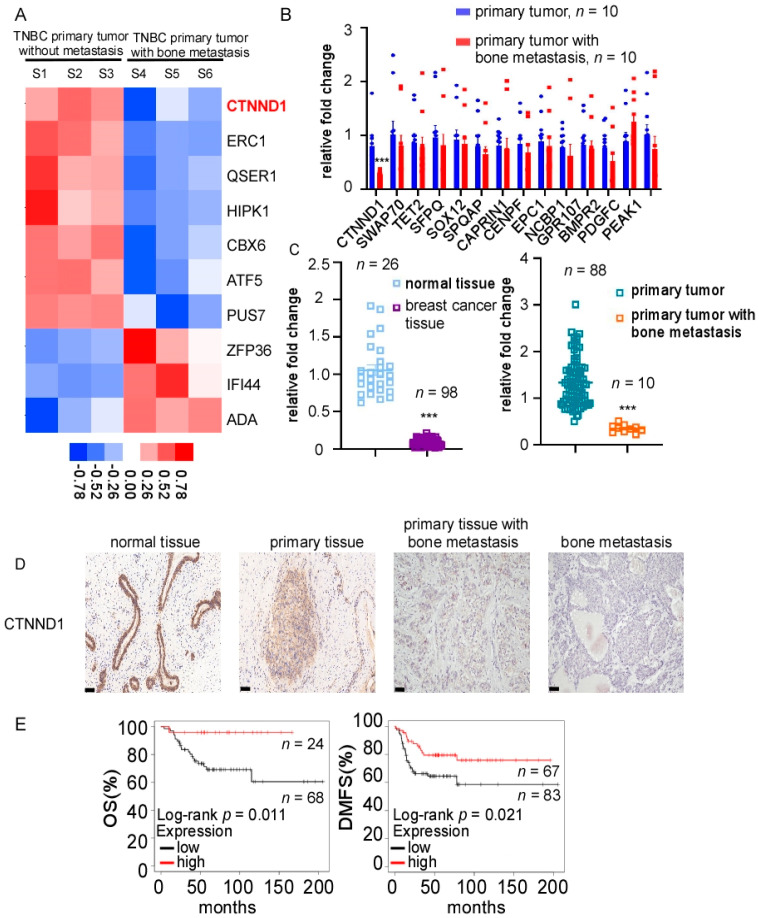
Identification of CTNND1 in TNBC with bone metastasis. (**A**) CTNND1 expression profile in TNBC with bone metastasis by high-throughput sequencing. Samples 1–3: TNBC without metastasis, samples 4–6: TNBC with bone metastasis. (**B**) Quantitative analysis of bone metastasis-related genes among the top one-hundred differentially expressed genes of high-throughput sequencing in primary TNBC tumor tissues without metastasis (*n* = 10) and with bone metastasis (*n* = 10). (**C**) Quantitative analysis of CTNND1 in normal breast tissues (*n* = 26), breast cancer tissues (*n* = 98), primary TNBC tumor tissues without metastasis, primary tumor tissues with bone metastasis (*n* = 10) by qRT-PCR. (**D**). The expression of CTNND1 in TNBC tissues by IHC. Scale bar represents 50 μm. (**E**) Kaplan–Meier analysis of the correlation between CTNND1 expression and overall survival (OS), distant metastasis-free survival (DMFS). All experiments were repeated at least 3 times. *** *p* < 0.005. Error bars indicate Standard Error of Mean (S.E.M.).

**Figure A1:**
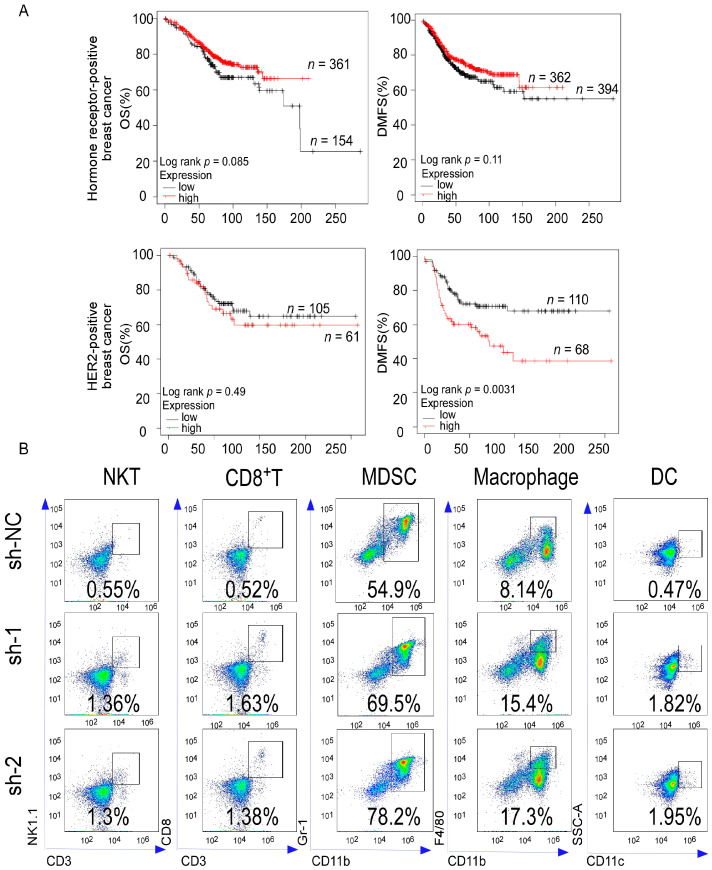
(**A**) Kaplan–Meier analysis of the correlation between CTNND1 expression and overall survival (OS), distant metastasis-free survival (DMFS) in other types of breast cancer (hormone receptor-positive breast cancer and HER2-positive breast cancer). (**B**) The infiltration of immune cells in the bone of mice with EO771 cells that had downregulated expressing of CTNND1 (EO771^CTNND1KD^)). All experiments were repeated at least 3 times. Error bars indicate Standard Error of Mean (S.E.M.).

To investigate the molecular profile of metastatic bone lesions derived from TNBC, high-throughput sequencing was performed in cohort 1, which included metastatic bone lesions from three patients who had only bone metastases and three early TNBC patients without any signs of distant metastasis. The top ten differentially expressed genes were shown in the heatmap (Figure 1A). Fourteen bone-metastasis-related genes in the top 100 differentially expressed genes were selected and quantified in cohort 2, which included ten TNBC patients with only bone-metastasis and ten early TNBC patients. Compared with early TNBC, CTNND1 was significantly downregulated in primary tumors of TNBC patients within the bone-metastasis subgroup (Figure 1B). We further quantified the expression level of fresh-frozen samples in cohort 3 and found that CTNND1 was decreased in TNBC tissues compared with normal tissues, and further decreased in primary TNBC tissues with bone metastasis (Figure 1C). We further detected the protein level of CTNND1 through IHC staining and found that the CTNND1 protein expression level was in interesting descending order with the highest seen in normal breast tissues, followed by in primary TNBC tissues without metastasis, then in TNBC primary tissues with bone-metastasis, with the lowest seen in metastatic bone tissues (Figure 1D). Clinical data from TCGA were analyzed which revealed that TNBC patients with lower CTNND1 expression had shorter disease-free survival (DFS) (*p* = 0.011) and shorter disease metastasis-free survival (DMFS) (*p* = 0.021) (Figure 1E). However, the expression of CTNND1 had no prominent influence on either OS or DMFS in other types of breast cancer (Figure A1A).

### 3.2. Decreased CTNND1 Promotes TNBC Bone Metastasis and Neutrophils Infiltration in Bone

**Figure 2:**
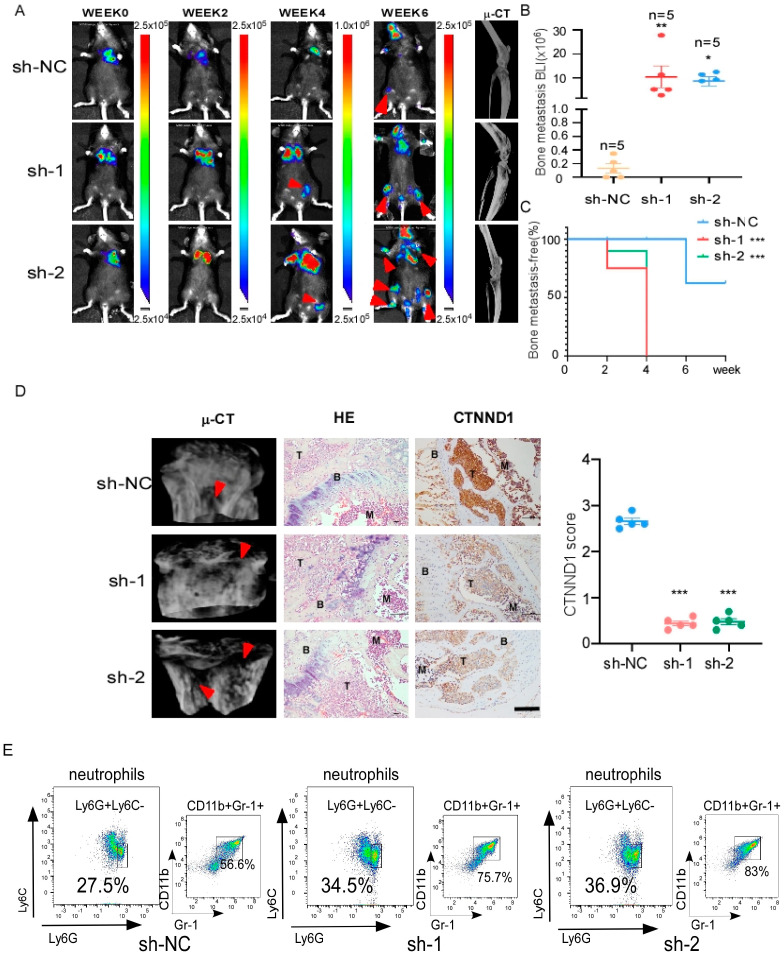
Downregulation of CTNND1 promotes bone metastasis of luciferase-labeled EO771 and immature myeloid cells, especially neutrophils infiltration in bone. (**A**) Representative BLI and μCT images of bone metastasis in mice. Arrowheads: bone metastasis of tumor cells. (**B**) In vivo quantification of bone metastasis in mice injected with EO771 cells of sh-control, sh-1, sh-2 by BLI (*** *p* = 0.004, * *p* = 0.0215). (**C**) Kaplan–Meier bone metastasis-free survival curve (*n* = 5/group). (**D**) μCT images of bone metastasis in mice (left), HE staining of bone metastasis tissues in mice (middle), the expression of CTNND1 in bone metastasis tissues by IHC staining (right). BMD (bone mineral density) and IHC scores were analyzed in the right graphs. Scale bar represents 50 μm, T: tumor, B: bone, M: bone marrow. (**E**) Representative flow cytometric analysis and quantification of mice bone for infiltrating immature myeloid cells and neutrophils in mice. All experiments were repeated at least 3 times. * *p* < 0.05, ** *p* < 0.01, *** *p* < 0.005. Error bars indicate Standard Error of Mean (S.E.M.).

To elucidate the function of CTNND1 in vivo, we monitored the development and progression of bone metastasis by bioluminescence signal (BLI) after intracardiac injection of the CTNND1 knockdown EO771^CTNND1KD^ or sh-control EO771^sh-controlD^ cells. Prominently, EO771^CTNND1KD^ displayed earlier bone metastatic onsets. Mice with high bone metastatic tumor burden had shorter animal survival (Figure 2A–C). Meanwhile, H&E and IHC staining were applied to confirm that knocking down CTNND1 (EO771^CTNND1KD^) accelerated TNBC bone metastasis (Figure 2D). Furthermore, μCT analysis revealed that decreased CTNND1 contributed to more severe osteolytic bone lesions (Figure 2D). In order to characterize the immune cells in the bone microenvironment, we analyzed the leukocyte fraction (CD45^+^ cells) by flow cytometry and found that there was a significant increase in immature myeloid cells infiltration, especially neutrophils in bone with EO771^CTNND1KD^ cell line (Figure 2E and Figure A1B).

### 3.3. CTNND1 Mediatesthe Migration and Invasion in TNBC Cells

**Figure 3:**
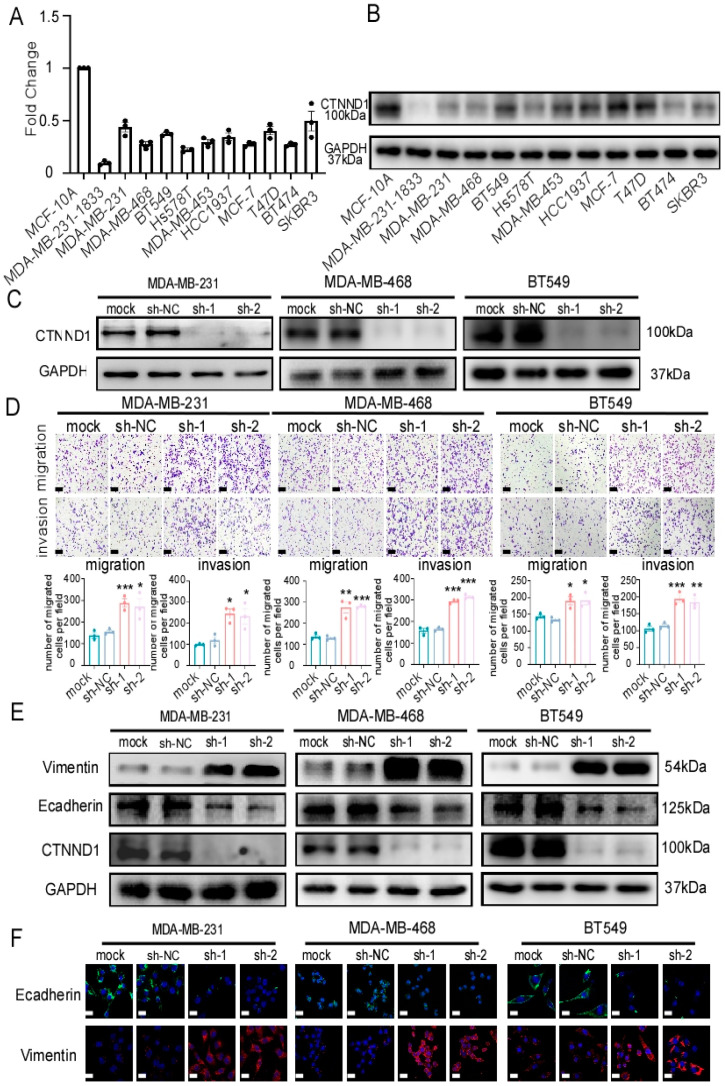
Downregulation of CTNND1 enhances EMT in TNBC cells. (**A**) CTNND1 expression level in human normal breast epithelial cells (MCF-10A) and various breast cancer cells (MDA-MB-231-1833, MDA-MB-231, MDA-MB-468, BT549, Hs578T, MDA-MB-453, HCC1937, MCF-7, T47D, BT474, SKBR3) by qRT-PCR. (**B**) Immunoblotting of the protein encoded by CTNND1 in human breast normal cell and breast cancer cells as the same as cell lines detected by qRT-PCR. (**C**) Immunoblotting of verifying knocking down CTNND1 stably in human TNBC cell lines (MDA-MB-231, MDA-MB-468, BT549). (**D**) Images and quantification of transwell migrated and invaded cells after CTNND1 knockdown. Scale bar represents 200 μm. (**E**) Immunoblotting of EMT markers in cells with CTNND1 knockdown or not. (**F**) Immunofluorescence of EMT markers in cells with CTNND1 knockdown or not. Scale bar represents 50 μm. All experiments were repeated at least 3 times. * *p* < 0.05, ** *p* < 0.01, *** *p* < 0.005. Error bars indicate Standard Error of Mean (S.E.M.). Please see the supplementary file for the original western blots used to assemble the figures.

**Figure A2:**
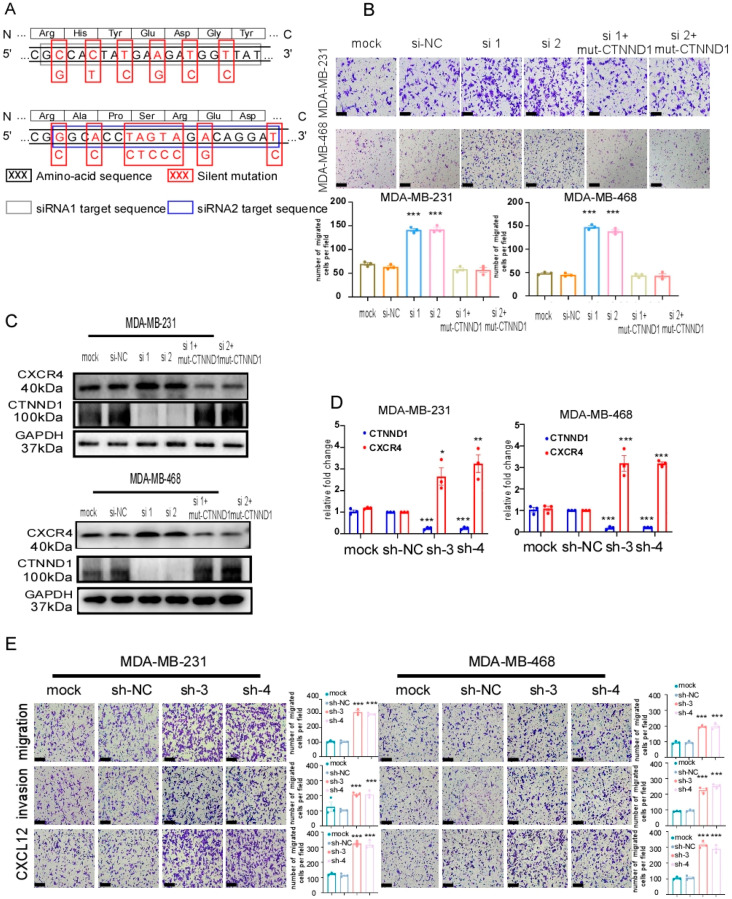
(**A**) Cartoon exemplifying the silent mutations in the siRNA1 and siRNA2 target sequence of WT CTNND1 cDNA, which confer resistance to the siRNA1 and siRNA2 and no change in the amino-acid sequence of the resulting protein (mut-CTNND1), compared with the WT. (**B**) Images and quantification of chemotaxis of MDA-MB-231 and MDA-MB-468 cells expressing mut-CTNND1 despite co-transfection with siRNA1 and siRNA2. (**C**) Immunoblot of CTNND1 and CXCR4 in MDA-MB-231 and MDA-MB-468. Note the strong expression of CTNND1 in cells expressing mut-CTNND1 despite co-transfection with siRNA1 and siRNA2. (**D**) Expression of CTNND1 and CXCR4 by qRT-PCR in MDA-MB-231 and MDA-MB-468 transfected with the other two sh-CTNND1 plasmids. (**E**) Images and quantification of transwell migrated and invaded cells after CTNND1 knockdown by being transfected with the other two sh-CTNND1 plasmids. Scale bar represents 200 μm. All experiments were repeated at least 3 times. * *p* < 0.05, ** *p* < 0.01, *** *p* < 0.005. Error bars indicate Standard Error of Mean (S.E.M.).

**Figure A3:**
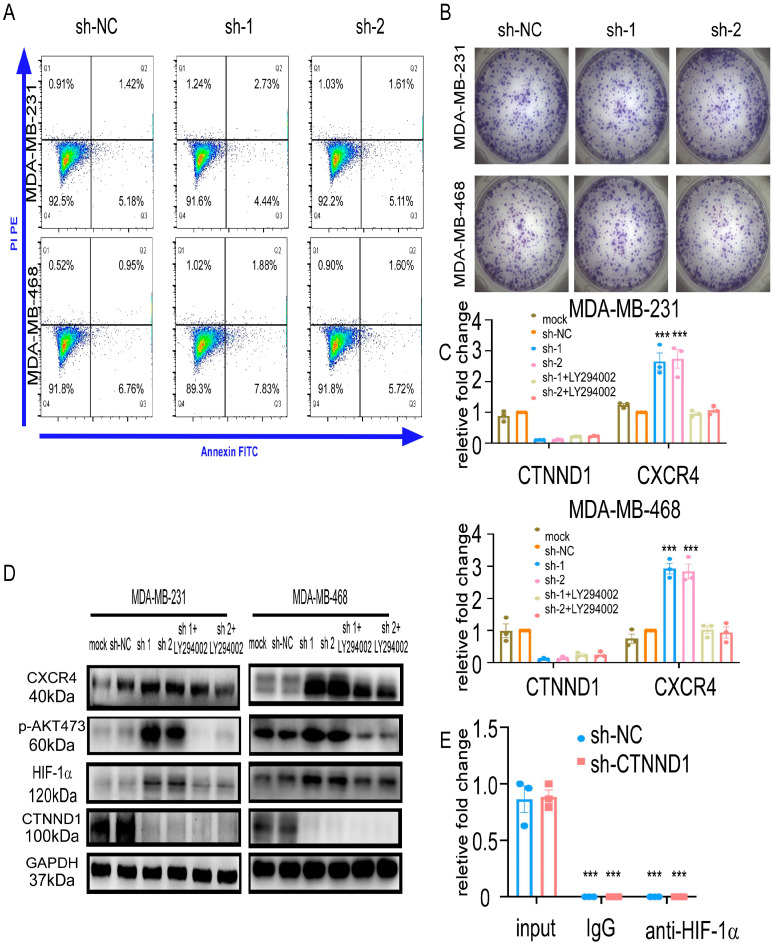
(**A**) The apoptosis of MDA-MB231, MDA-MB-468 with downregulation of CTNND1 analyzed by flow cytometry. (**B**) The colony formation of MDA-MB231, MDA-MB-468 with downregulation of CTNND1. (**C**) Expression of CTNND1 and CXCR4 in MDA-MB231 and MDA-MB-468 with CTNND1 reduction and PI3K inhibitor LY294002 by qRT-PCR. (**D**) Immunoblotting of the activation PI3K/AKT/HIF-1α pathway with downregulation of CTNND1 or LY294002 in MDA-MB231 and MDA-MB-468 cells. (**E**) The enrichment of β-actin in MDA-MB-231 cells transduced with sh-NC or sh-CTNND1 by CHIP-qPCR. All experiments were repeated at least 3 times. *** *p* < 0.005. Error bars indicate Standard Error of Mean (S.E.M.).

We further validated the expression of CTNND1 in cell lines and found that breast epithelial cell line MCF-10A expressed higher CTNND1 than breast cancer cell lines in which bone metastatic MDA-MB-231-1833 cell line (an MDA-MB-231 variant with high metastatic bone tropism) displayed the lowest CTNND1 expression (Figure 3A,B). To explore how CTNND1 regulates bone metastasis, we first constructed TNBC cell lines stably knocking down CTNND1 (Figure 3C and Figure A2D) and investigated its effect on the intrinsic malignancy of tumor cells. Knockdown of CTNND1 enhanced cell migration and cell invasion (Figure 3D and Figure A2E). Meanwhile, knockdown of CTNND1 regulated the expression of epithelial–mesenchymal transition (EMT)-related markers (E-cadherin, Vimentin) (Figure 3E,F). In addition to the metastasis-related phenotype, we found that CTNND1 had no effect on cell growth and apoptosis of TNBC cells (Figure A3A,B).

### 3.4. CTNND1 Downregulation Promotes TNBC Recruitment to Bone via Upregulating CXCR4

**Figure 4:**
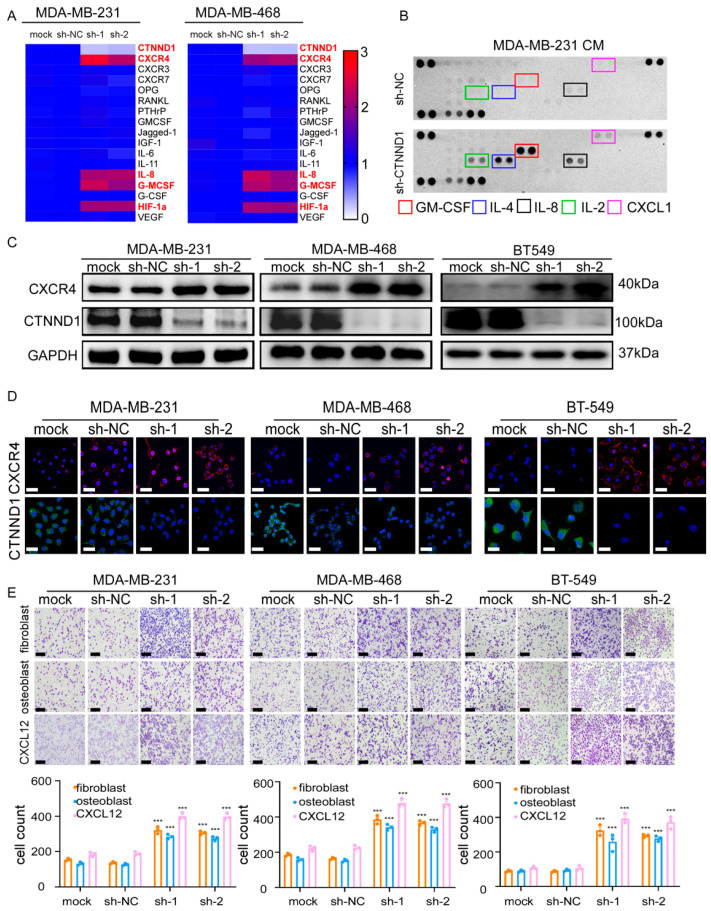
Reduction of CTNND1 upregulates the CXCR4/CXCL12 axis to facilitate TNBC cells homing to the bone. (**A**) Expression of bone metastasis-related genes was detected by qRT-PCR in human TNBC cell lines (MDA-MB-231, MBA-MB-468) with and without CTNND1 knockdown. The result was showed as a heatmap. (**B**) Cytokines secreted by MDA-MB-231 with CTNND1 knockdown were detected by human Cytokine Array Kit. (**C**) Immunoblotting of CXCR4 in TNBC cells with CTNND1 knockdown. (**D**) Immunofluorescence of CXCR4 in TNBC cells with CTNND1 knockdown. Scale bar = 50 μm. (**E**) Images and quantification of chemotaxis of TNBC cells with CTNND1 knockdown, fibroblast (upper panel), osteoblast (middle panel), CXCL12 (lower panel) in bottom chamber, respectively. Scale bar = 200 μm. *** *p* < 0.005. Error bars indicate Standard Error of Mean (S.E.M.).

Since decreased CTNND1 promoted the intrinsic malignancy of breast cancer cells and correlated more frequently with bone metastasis in breast cancer patients, we next explored whether CTNND1 plays a role in bone metastasis of breast cancer. We firstly detected the expression of bone metastasis related genes by real-time PCR and found that downregulation of CTNND1 significantly upregulated CXCR4, GM-CSF, IL-8 and HIF-1α in both MDA-MB-231 and MDA-MB-468 cell lines (Figure 4A). We then compared the difference of cytokines secreted in the conditioned medium (CM) of MDA-MB-231 transduced with sh-NC orsh-CTNND1 and found that the expression levels of GM-CSF, IL-8, IL-4, IL-2 and CXCL1 were higher in CM of cells with reduced CTNND1 than that of control cells (Figure 4B). In TNBC cell lines, we further validated that knockdown of CTNND1 upregulated CXCR4 (Figure 4C,D). To avoid the off-target effect of siRNA, we performed RNAi-resistant experiments and confirmed that CTNND1 regulated CXCR4 (Figure A2A,C). As the CXCR4/CXCL12 axis was well known for chemotaxis of tumor cells in bone metastasis [24,25], we co-cultured TNBC cell lines with reduced CTNND1 together with fibroblasts, osteoblasts and CXCL12 cytokine, respectively, as CXCL12 was mainly produced by fibroblasts and osteoblasts in bone. Indeed, knockdown of CTNND1 accelerated TNBC cells migration through upregulation of the CXCR4/CXCL12 axis (Figure 4E and Figure A2B,E).

### 3.5. Knocking Down CTNND1 Upregulates CXCR4 via PI3K/AKT/HIF-1α Pathway

**Figure 5:**
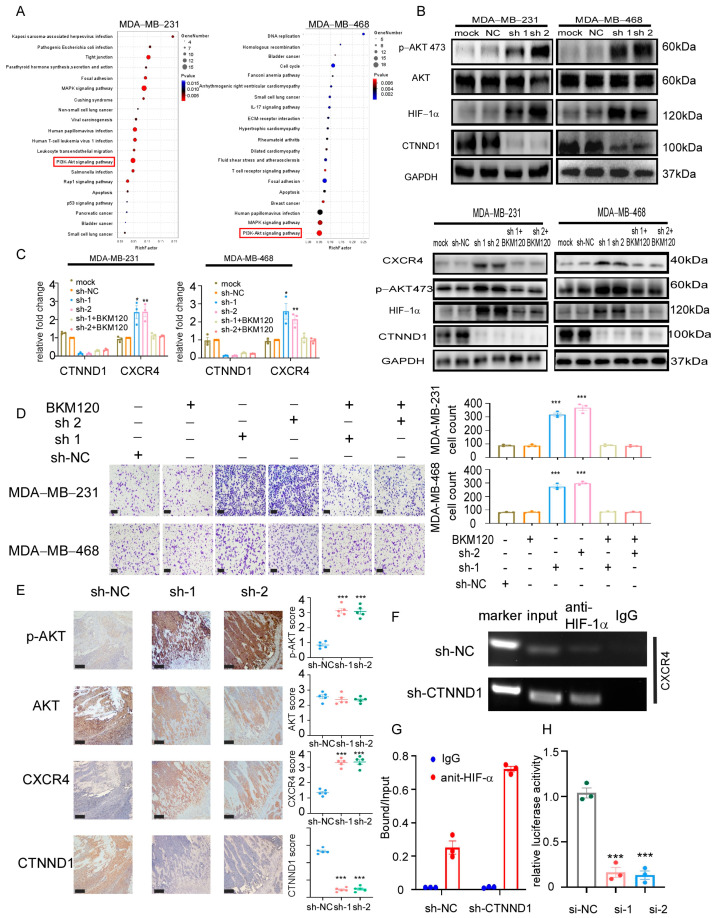
Knockdown of CTNND1 upregulates CXCR4 via activation of PI3K/AKT/HIF-1α pathway. (**A**) KEGG pathway analysis of RNA sequencing between cells with sh-NC and sh-CTNND1 in MDA-MB-231 and MDA-MB-468 cells. (**B**) Immunoblotting of verifying activation of PI3K/AKT/HIF-1α pathway. (**C**) Expression of CXCR4 by qRT-PCR (left) and immunoblotting (right) in cells with CTNND1 knockdown or BKM120. (**D**) Images and quantification of chemotaxis of cells with CTNND1 knockdown or BKM120 in the upper chamber and CXCL12 in the bottom chamber. Scale bar: 200 μm. (**E**) IHC staining of AKT, p-AKT and CXCR4 in the bone of mice withEO771^CTNND1KD^. Scale bar: 200 μm, T: tumor, B: bone, M: bone marrow. (**F**) Chromatin immunoprecipitation (ChIP) assay of the enrichment of HIF-1α at the CXCR4 promoter relative to IgG in MDA-MB-231 cells transduced with sh-CTNND1. (**G**) The enrichment of HIF-1α at the CXCR4 promoter relative to Input in MDA-MB-231 cells transduced with sh-CTNND1 by CHIP-qPCR. (**H**) Luciferase reporter assay for the CXCR4 promoter construct transfected in MDA-MB-231 cells. * *p* < 0.05, ** *p* < 0.01, *** *p <* 0.005. Error bars indicate Standard Error of Mean (S.E.M.).

To elucidate the underlying mechanisms by which knockdown CTNND1 upregulated CXCR4, we performed transcriptome sequencing analysis in MDA-MB-231 and MDA-MB-468 cells with CTNND1 knockdown cells. Combined with Kyoto Encyclopedia of Genes and Genomes (KEGG) database pathway analyses, PI3K/AKT pathway was found to be activated in both cancer cell lines when their CTNND1 were knocked down (Figure 5A). We next screened the differentially expressed genes (compared with the control cells) and then overlapped with the previously reported bone metastasis-related genes that correlated with PI3K/AKT pathway. We found that knockdown of CTNND1 activated the PI3K/AKT pathway and also upregulated HIF-1α (Figure 5B). To verify the activation of the PI3K/AKT pathway in vivo experiments, we examined its activation via IHC staining in the bone of mice bearing tumor with CTNND1 knockdown (Figure 5E). The mRNA of CXCR4 was prominently upregulated in cancer cells with CTNND1 knockdown, suggesting that it might be regulated at the transcription level. We further demonstrated that the PI3K inhibitor BKM120 and LY294002 were able to abolish the upregulation of CXCR4 and HIF-1α seen in cells with CTNND1 knockdown (Figure 5C and Figure A3C,D). Moreover, the PI3K/AKT inhibitors strongly suppressed chemotaxis of TNBC cells, likely due to the concurrent inhibition of the CXCR4/CXCL12 axis (Figure 5D). HIF-1α chromatin immunoprecipitation (ChIP), followed by quantitative PCR (qPCR), demonstrated that knockdown of CTNND1 enhanced the binding of HIF-1α to the CXCR4 transcription start site in MDA-MB-231 cells (Figure 5F,G and Figure A3E). Knockdown of HIF-1α prominently abolished luciferase activity in MDA-MB-231 transfected with CXCR4 promoter construct (Figure 5H).

### 3.6. Neutrophils Infiltration Promotes Survival of the Downexpressing-CTNND1 Cells in Bone

**Figure 6:**
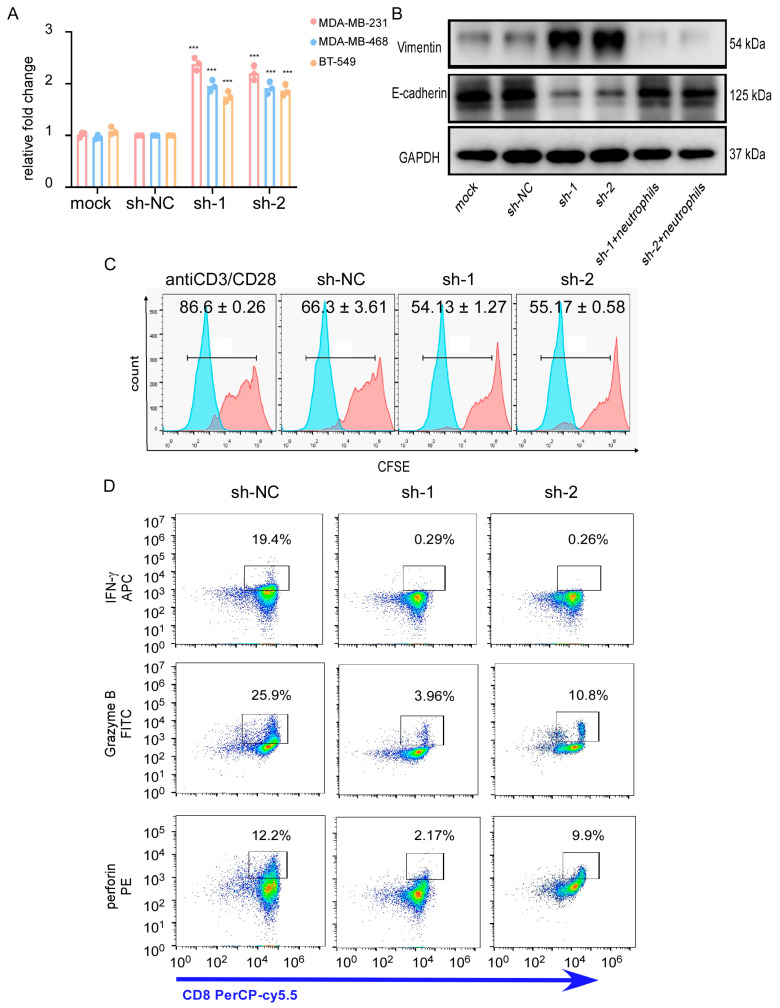
Neutrophil infiltration promotes survival of tumor cells’ lack of CTNND1 expression via impairing cytotoxicity of CTLs. (**A**) Recruitment of neutrophils was detected through CKK8 with neutrophils in the upper chamber (core size 5 μm) and cells (MDA-MB-231, MDA-MB-468, BT549) with CTNND1 knockdown in the bottom chamber cocultured for 1 h. (**B**) Immunoblotting of EMT markers were detected in EO771CTNND1KD cocultured with neutrophils isolated from bone metastasis of mice with EO771^CTNND1KD^. (**C**) Proliferation of CFSE-labeled CTLs was detected by flow cytometry after being cocultured with neutrophils isolated from the bone metastasis of mice for 48 h. mean ± s.d., *n* = 3. (**D**) Expression of granzyme B, IFN-γ, perforin was detected via flow cytometry in CTLs cocultured with neutrophils isolated from bone metastasis of mice with or without CTNND1 knockdown. *** *p* < 0.005. Error bars indicate Standard Error of Mean (S.E.M.).

As we mentioned before, knockdown of CTNND1 enhanced bone metastasis of TNBC cells and accelerated neutrophils infiltration in bone. In addition to CXCR4, knockdown of CTNND1 also upregulated GM-CSF and IL-8, cytokines known to function as powerful regulators of immune infiltration [27,28]. Thus, we hypothesized that upregulation of GM-CSF and IL-8 in TNBC cells with CTNND1 knockdown might promote cells’ colonization or survival via remodeling bone immune microenvironment. We firstly found that CM from CTNND1 knockdown human TNBC cells enhanced the recruitment of neutrophils isolated from blood (Figure 6A). Then, we co-cultured, in vitro, TNBC EO771 cells with neutrophils isolated from bone marrow of C57BL/6 mice that displayed bone metastasis. It was shown that neutrophils from mice promoted EO771 cells mesenchymal-epithelial transition (MET) (Figure 6B). We further explored whether neutrophils might influence immune effector cells, especially cytotoxic T cells to promote cells survival in vitro. We isolated neutrophils from C57BL/6 mice with bone metastasis of CTNND1 knockdown EO771 cells and co-cultured with cytotoxic T cells. We found that cytotoxic T cells proliferation and production of perforin, granzyme B and IFN-y were significantly inhibited (Figure 6C,D). These findings collectively suggested that knockdown of CTNND1 might facilitate cell survival of TNBC cells in bone through upregulating GM-CSF and IL-8 and accelerated neutrophils infiltration to inhibit cytotoxic T cells responses.

## 4. Discussion

In this study, by way of high-throughput sequencing, we identified CTNND1 downregulation in TNBC patients with only bone metastasis. Clinically, TNBC patients with lower CTNND1 were associated with a higher rate of bone metastasis and shorter OS, DMFS. In vivo, knockdown of CTNND1 accelerated TNBC cells bone metastasis and the infiltration of immature myeloid cells, especially neutrophils in bone. Mechanistically, knockdown of CTNND1 promoted tumor cells EMT and homing to the bone via upregulation PI3K/AKT/HIF-1α/CXCR4 pathway. When recruited to the bone, tumor cells with CTNND1 reduction exhibit a stronger ability to survive by secreting more GM-CSF and IL-8, which contributed to the infiltration of immature myeloid cells, especially neutrophils, impairing proliferation and cytotoxicity of CTLs.

Previous studies revealed that p120^ctn^, the protein encoded by CTNND1, had fundamental roles in cell biology. It was expressed in various types of cells and required for normal function of these cells [10,12]. In recent years, researchers had discovered that the dysregulation of CTNND1 impaired the homeostasis in vivo, especially in tumor [12]. A recent study found that p120-ablation apparently accelerated pro-metastasis of tumor cells in p120 knockout (KO) PyMT mouse model. However, pulmonary metastases from p120-KO mice were strongly p120-positive, which seemed contradictory to the findings in the present study [29]. A plausible explanation for the discrepancy is the variation of the models used in the respective studies. The p120-KO mice might construct a totally different microenvironment when compared to our animal model in which only injected breast cancer cells (EO771^CTNND1KD^) received modification of the CTNND1, where the host microenvironment remains unaffected. Furthermore, these studies examined metastases at different destinations, namely lung versus bones. Under different metastatic destinations, the heterogeneity of tumor cells in metastatic bone lesions and in pulmonary metastases are different in a different bone and lung environments. Here, we focused on the role of CTNND1 in TNBC bone metastasis and explored its underlying mechanism. There were several interesting findings in our study that could be potentially turned into clinical transformation. Firstly, TNBC patients with bone metastasis always received delay treatments because it could not be detected early and accurately enough. We verified that reduction of CTNND1 accelerated bone metastasis of TNBC, suggesting that CTNND1 might be a biomarker for early identifying the TNBC patients with high risk of developing bone metastasis and then improved the clinical outcome of these patients. With the development of DNA-microarray and liquid biopsy detection, CTNND1 might be a promising gene biomarker if it could be dynamically monitored at the circulating tumor DNA level, which promoted CTNND1 to be detected conveniently in clinics [30]. Secondly, we found that upregulation of CXCR4 in CTNND1 knockdown TNBC cells can be reversed by PI3K inhibitors. Thus, drugs or molecules that targeted the PI3K/AKT pathway might be taken into account in TNBC patients with high risk of bone metastasis or TNBC patients who had clinically detectable bone metastasis. Thirdly, we found that CTNND1 knockdown in TNBC cells resulted in more infiltration of immature myeloid cells, especially neutrophils in bone microenvironment, and CTLs in bone could not perform its cytotoxic function well. This finding provided tentative evidence for the use of immunotherapy in TNBC patients with bone metastasis. Combination with immunotherapy might improve the therapeutic effect of TNBC patients who were not sensitive to traditional anticancer therapy.

Currently, immune checkpoint inhibitors have led to a surge in new investigational therapies for the treatment of solid tumors. However, current standard immunotherapies targeting T cell activation have been less reliable for TNBC patients with bone metastasis. In addition, the contributions of immune microenvironment to the clinical cause of TNBC patients with bone metastasis are less clear and less extensively studied. Given the abundance of immune cells in the bone microenvironment, there is a significant need for defining the role of immune populations. It was reported recently that CTSC (cathepsin C) secretion promoted lung metastasis of breast cancer via facilitating neutrophils extracellular traps (NETs) formation [31]. In the liver metastasis of breast cancer, the transmembrane CCDC25 acted as a NET-DNA receptor on cancer cells to interact with NET and enhance cell motility [32]. Thus, it would be worthwhile to further explore the function of neutrophils infiltrated in bone with metastasis of TNBC cells that have CTNND1 downregulation. In our study, reduction of CTNND1 in TNBC cells facilitated immature myeloid cells, especially neutrophils infiltration in bone through secreting more GM-CSF and IL-8. The infiltrated neutrophils might enhance the growth of tumor cells in bone directly through NETs or indirectly by forming immunosuppressive microenvironment such as weakening cytotoxicity of CTLs. Taken together, a better understanding of the immune microenvironment in bone might contribute to discover a new treatment.

## 5. Conclusions

Reduction of CTNND1 promotes bone metastasis of TNBC cells by facilitating homing cancer cells to bone and the survival of the metastatic cells via upregulating CXCR4/CXCL12 axis and neutrophils Infiltration in bone. CTNND1 may be a biomarker to early and accurately predict the risk of the bone metastasis in TNBC patients.

## Data Availability

The data presented in this study are available upon request from the corresponding authors.

## Supplementary Materials

The following supporting information can be downloaded at: https://www.mdpi.com/article/10.3390/cancers13225703/s1. Supplementary File S1: Original Western blots used to assemble the figures shown in the manuscript; Figures S2–S6.

## Appendix A

**Table A1:** Nucleotide sequence of the primers used for qRT-PCR.

Gene	Species	Forward Primer (5′–3′)	Reverse Primer (3′–5′)
β-actin	Homo Sapiens	AGCGGGAAATCGTGCGTGAC	CAGGAAGGAAGGCTGGAAGAGT
CXCR4	Homo Sapiens	TCGTCCACGCCACCAA	GCAGGATAAGGCCAACCAT
CTNND1	Homo Sapiens	GCATTGAGGAGCGGTAT	CGAGGGTCAGAGGGTGT
IL-8	Homo Sapiens	ACATACTCCAAACCTTTCCACC	AAAAACTTCTCCACAACCCTCT
GM-CSF	Homo Sapiens	GGTCATCTTGGAGGGACCAA	CCATGCCTGTATCAGGGTCAG
G-CSF	Homo Sapiens	GCTGCTTGAGCCAACTCCATA	GAACGCGGTACGACACCTC
VEGF	Homo Sapiens	ATCTCTCACCAGGAAAGACTGA	AAGAAAAATAAAATGGCGAATC
IL-6	Homo Sapiens	GGGAGAGGGAGCGATAAACA	AGAAGGCAACTGGACCGAAG
HIF-1α	Homo Sapiens	AAGACACAGAAGCAAAGAACCC	ACTCAAAGCGACAGATAACACG
CXCR7	Homo Sapiens	GTCTGCGTCCAACAATGAGA	GAACGGCAAAGCCCAAG
RANKL	Homo Sapiens	GAAATGCTGGGGTGGTAG	GGCAGGAATGACGGTAAA
IL-11	Homo Sapiens	GCTGCAAGGTCAAGATGGTT	GGCTGGGTGGCGTTCTA
IGF-1	Homo Sapiens	TTTGCCCTCTGCCTGTTT	GCATCTTTCAGCTTTCCTCC
PTHrP	Homo Sapiens	TTACGGCGACGATTCTTCCT	CTCCACCTTGTTAGTTTCCTGAGTT
Jagged-1	Homo Sapiens	TAATGGTTATCGCTGTATCTGTCC	AGTTCTTGCCCTCATAGTCCTC
OPG	Homo Sapiens	GTGTGCGAATGCAAGGAAGG	CCACTCCAAATCCAGGAGGG
CXCR3	Homo Sapiens	CCACCTAGCTGTAGCAGACAC	AGGGCTCCTGCGTAGAAGTT
TNF-α	Homo Sapiens	ATGTCTGGCACATGGAAGGTG	GGAACTGTTGGGGAGAAGGAG
SWAP70	Homo Sapiens	GTGGCTGCGGAGGTTGAG	CCTTGAGCTGGGACTTGGAG
TET2	Homo Sapiens	GCAGCACACCCTCTCAAGAT	GTCCTATTGGCTGCTGCTGA
SFPQ	Homo Sapiens	AGCGATGTCGGTTGTTTGTTG	AGCGAACTCGAAGCTGTCTAC
SOX12	Homo Sapiens	CTGGAGTGGTGGGATTGGTC	GGGTGTCAGAGGGACAAAGG
SPQAP	Homo Sapiens	CCAGGTAAGAGCCCTGTTCA	AGGGGCGAGACAAGTAGAGA
CAPRIN1	Homo Sapiens	CACGTCGGGAGCAGCTTATG	CAGGTCAGTCCGCACTTCATC
CENPF	Homo Sapiens	TGGAAGCACTGATCACCTGT	CCCACATACAAACAGAGATTGTGA
EPC1	Homo Sapiens	TGGCAGTGGGAAAACCCTTT	AGGTGGCATAGGCAGCATTT
NCBP1	Homo Sapiens	GTTGCACGCCTATTACCTGAG	GGACCAAATACACGGCTTCATTA
GPR107	Homo Sapiens	AGGGCTTCCCTATCGAAGG	CCAGTGCCAATGAGTGCAAT
BMPR2	Homo Sapiens	GACAGGAGACCGTAAACAAGG	CCATATCGACCTCGGCCAATC
PDGFC	Homo Sapiens	TGAGCCTTCTCTGCCTCTTG	ACCATACCACCTATCACCAAGC
PEAK1	Homo Sapiens	TAGTGGCAGATGGGCAAAGTA	TGTTTCGGTTCCACCCTATGA
CTNND1	mouse	TCCTATCCTCCCAGACCCTAC	ATAAAAAGTTCCCCCCACACC
