# Correction: Lin et al. Silencing CTNND1 Mediates Triple-Negative Breast Cancer Bone Metastasis via Upregulating CXCR4/CXCL12 Axis and Neutrophils Infiltration in Bone. *Cancers* 2021, *13*, 5703

**DOI:** 10.3390/cancers17223674

**Published:** 2025-11-17

**Authors:** Qun Lin, Xiaolin Fang, Gehao Liang, Qing Luo, Yinghuan Cen, Yu Shi, Shijie Jia, Juanmei Li, Wenqian Yang, Andrew J. Sanders, Chang Gong, Wenguo Jiang

**Affiliations:** 1 Breast Tumor Center, Sun Yat-sen Memorial Hospital, Sun Yat-sen University, Guangzhou 510120, China; linq27@mail2.sysu.edu.cn (Q.L.); fangxlin3@mail.sysu.edu.cn (X.F.); lianggeh@mail2.sysu.edu.cn (G.L.); luoq63@mail2.sysu.edu.cn (Q.L.); cenyh@mail2.sysu.edu.cn (Y.C.); shiy77@mail2.sysu.edu.cn (Y.S.); jiashj3@mail2.sysu.edu.cn (S.J.); lijm95@mail.sysu.edu.cn (J.L.); yangwq29@mail.sysu.edu.cn (W.Y.); 2 Guangdong Provincial Key Laboratory of Malignant Tumor Epigenetics and Gene Regulation, Sun Yat-sen Memorial Hospital, Sun Yat-sen University, Guangzhou 510120, China; 3 Department of Breast Oncology, Sun Yat-sen University Cancer Center, Sun Yat-sen University, Guangzhou 510080, China; 4 Cardiff China Medical Research Collaborative, Cardiff University School of Medicine, Cardiff University, Heath Park, Cardiff CF14 4XN, UK; sandersaj1@cardiff.ac.uk

## Error in Figure

In the original publication [1], there was a mistake in Figures 4E, 5D and 6D as published. It has come to our attention that an error was made during assembly of these figures, resulting in the incorporation of incorrect representative images. The corrected Figure 4, Figure 5 and Figure 6 appear below. The authors state that the scientific conclusions are unaffected. This correction was approved by the Academic Editor. The original publication has also been updated.

## Update to Supplementary Materials

The corresponding original Western blot images have also been included in the Supplementary Materials file, which contains all original images used in the manuscript for the assembly of representative Western blot figures.

## Figures and Tables

**Figure 4:**
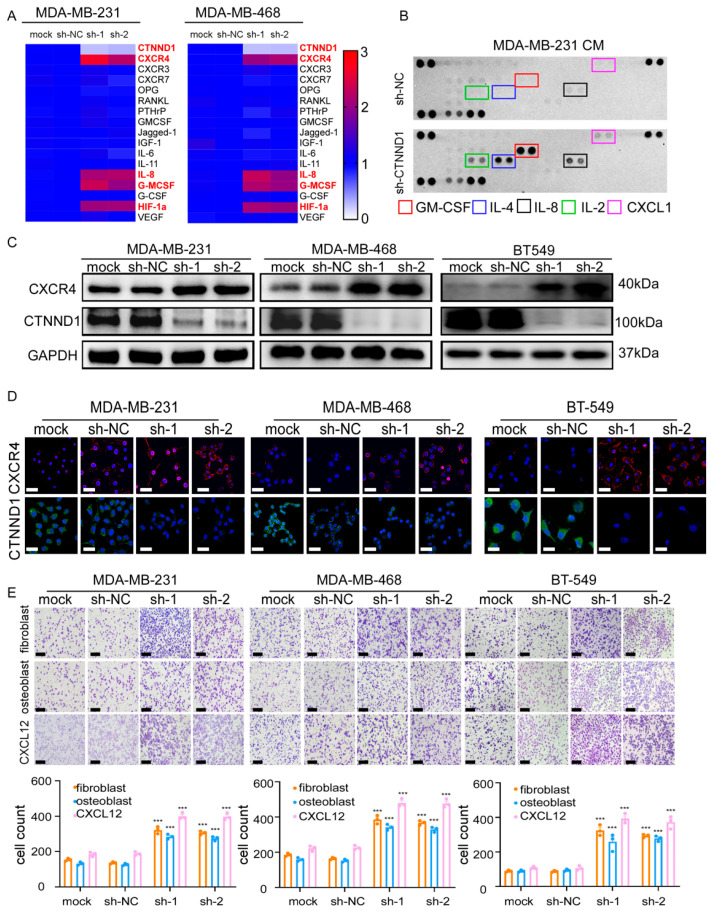
Reduction of CTNND1 upregulates the CXCR4/CXCL12 axis to facilitate TNBC cells homing to the bone. (**A**) Expression of bone metastasis-related genes was detected by qRT-PCR in human TNBC cell lines (MDA-MB-231, MBA-MB-468) with and without CTNND1 knockdown. The result was showed as a heatmap. (**B**) Cytokines secreted by MDA-MB-231 with CTNND1 knockdown were detected by human Cytokine Array Kit. (**C**) Immunoblotting of CXCR4 in TNBC cells with CTNND1 knockdown. (**D**) Immunofluorescence of CXCR4 in TNBC cells with CTNND1 knockdown. Scale bar = 50 μm. (**E**) Images and quantification of chemotaxis of TNBC cells with CTNND1 knockdown, fibroblast (upper panel), osteoblast (middle panel), CXCL12 (lower panel) in bottom chamber, respectively. Scale bar = 200 μm. *** *p* < 0.005. Error bars indicate Standard Error of Mean (S.E.M.).

**Figure 5:**
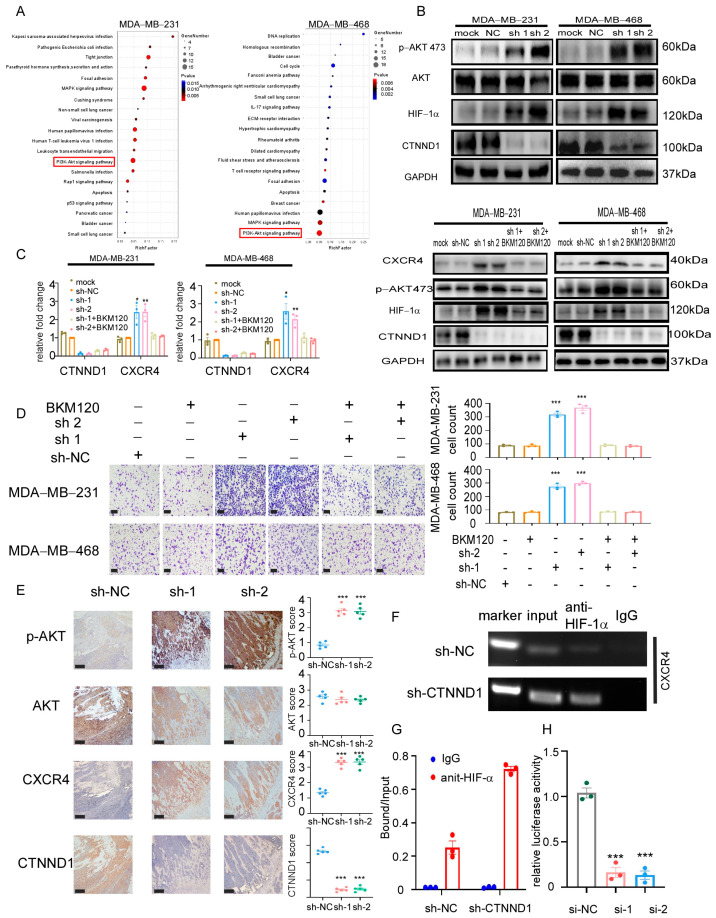
Knockdown of CTNND1 upregulates CXCR4 via activation of PI3K/AKT/HIF-1α pathway. (**A**) KEGG pathway analysis of RNA sequencing between cells with sh-NC and sh-CTNND1 in MDA-MB-231 and MDA-MB-468 cells. (**B**) Immunoblotting of verifying activation of PI3K/AKT/HIF-1α pathway. (**C**) Expression of CXCR4 by qRT-PCR (left) and immunoblotting (right) in cells with CTNND1 knockdown or BKM120. (**D**) Images and quantification of chemotaxis of cells with CTNND1 knockdown or BKM120 in the upper chamber and CXCL12 in the bottom chamber. Scale bar: 200 μm. (**E**) IHC staining of AKT, p-AKT and CXCR4 in the bone of mice withEO771^CTNND1KD^. Scale bar: 200 μm, T: tumor, B: bone, M: bone marrow. (**F**) Chromatin immunoprecipitation (ChIP) assay of the enrichment of HIF-1α at the CXCR4 promoter relative to IgG in MDA-MB-231 cells transduced with sh-CTNND1. (**G**) The enrichment of HIF-1α at the CXCR4 promoter relative to Input in MDA-MB-231 cells transduced with sh-CTNND1 by CHIP-qPCR. (**H**) Luciferase reporter assay for the CXCR4 promoter construct transfected in MDA-MB-231 cells. * *p* < 0.05, ** *p* < 0.01, *** *p* < 0.005. Error bars indicate Standard Error of Mean (S.E.M.).

**Figure 6:**
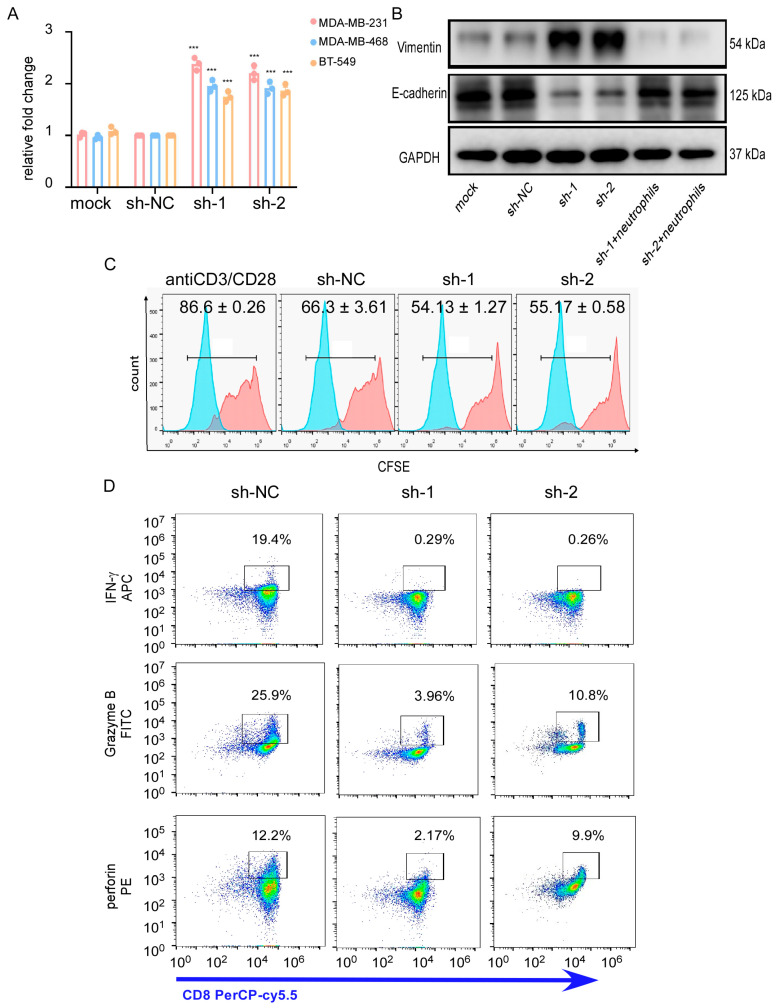
Neutrophil infiltration promotes survival of tumor cells’ lack of CTNND1 expression via impairing cytotoxicity of CTLs. (**A**) Recruitment of neutrophils was detected through CKK8 with neutrophils in the upper chamber (core size 5 μm) and cells (MDA-MB-231, MDA-MB-468, BT549) with CTNND1 knockdown in the bottom chamber cocultured for 1 h. (**B**) Immunoblotting of EMT markers were detected in EO771CTNND1KD cocultured with neutrophils isolated from bone metastasis of mice with EO771^CTNND1KD^. (**C**) Proliferation of CFSE-labeled CTLs was detected by flow cytometry after being cocultured with neutrophils isolated from the bone metastasis of mice for 48 h. mean ± s.d., *n* = 3. (**D**) Expression of granzyme B, IFN-γ, perforin was detected via flow cytometry in CTLs cocultured with neutrophils isolated from bone metastasis of mice with or without CTNND1 knockdown. *** *p* < 0.005. Error bars indicate Standard Error of Mean (S.E.M.).
