# Supplementary material for: Silencing CTNND1 Mediates Triple-Negative Breast Cancer Bone Metastasis via Upregulating CXCR4/CXCL12 Axis and Neutrophils Infiltration in Bone
Source: Cancers (Basel). 2021 Nov 17;13(22):5703. doi: 10.3390/cancers13225703 (PMC8616231; doi:10.3390/cancers13225703)
Supplement: Supplementary file 1 [file cancers-13-05703-s001.zip › western blots provided by authors.pdf]

Figure S3B

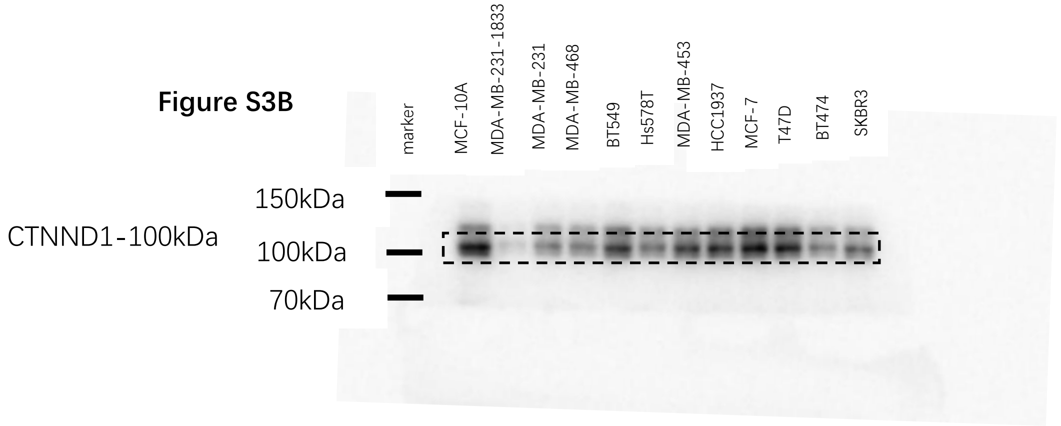

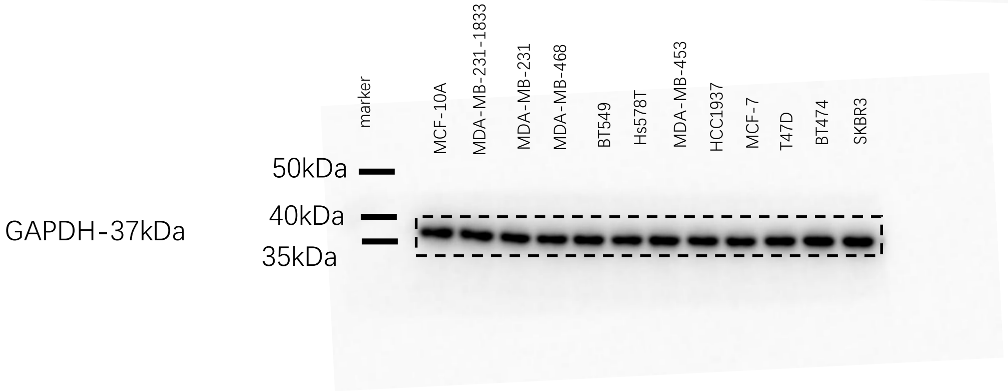

Figure S3C-MDA-MB-231

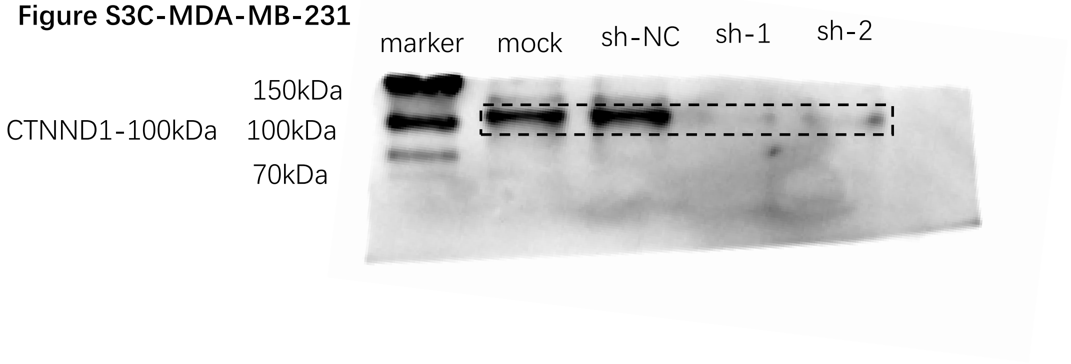

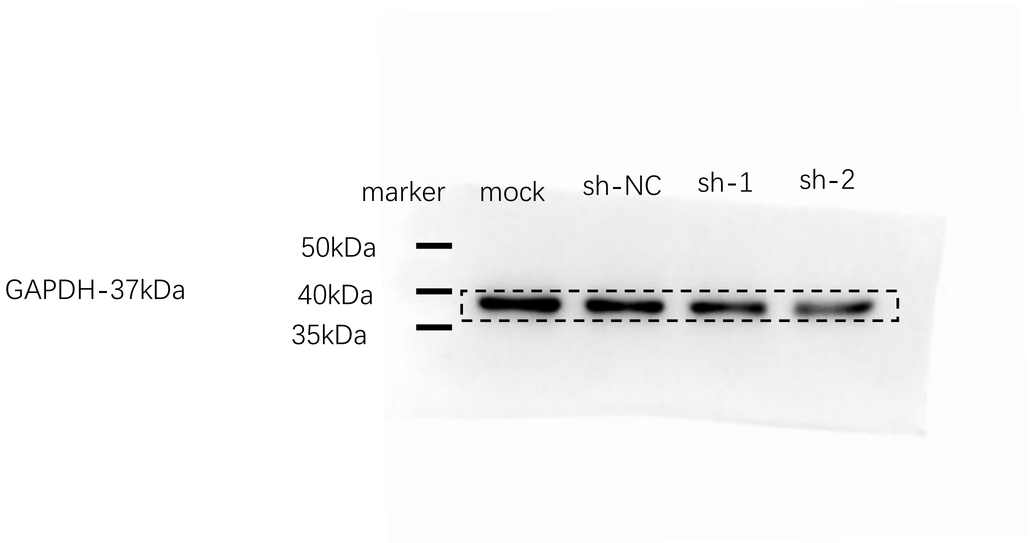

Figure S3C-MDA-MB-468

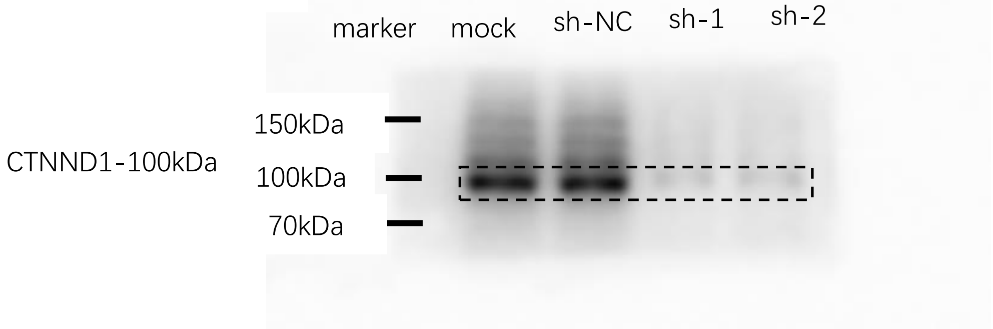

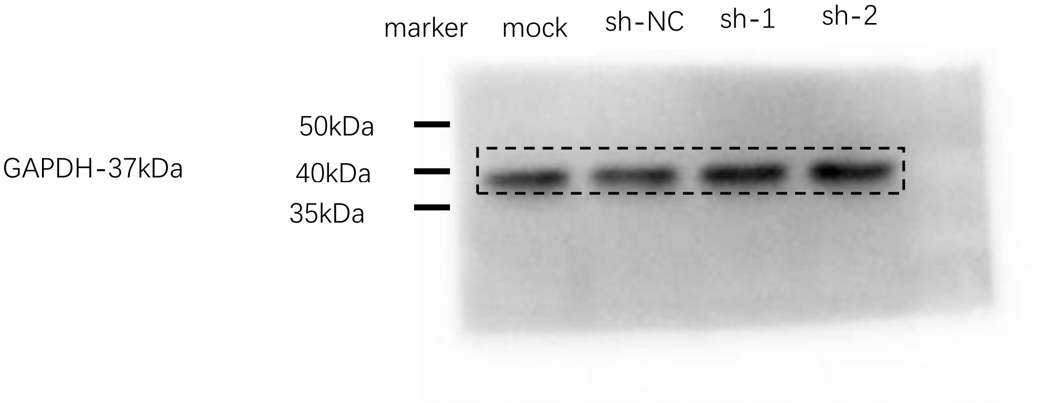

Figure S3C-BT549

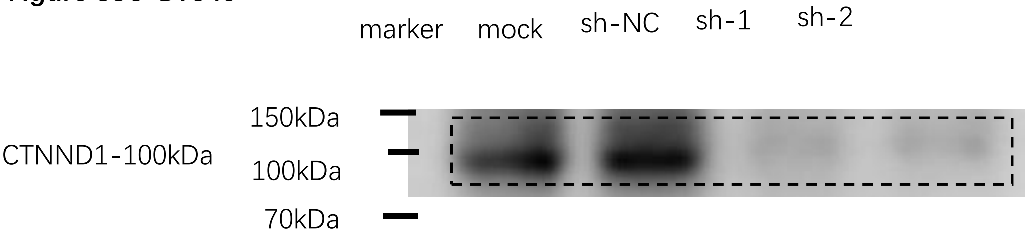

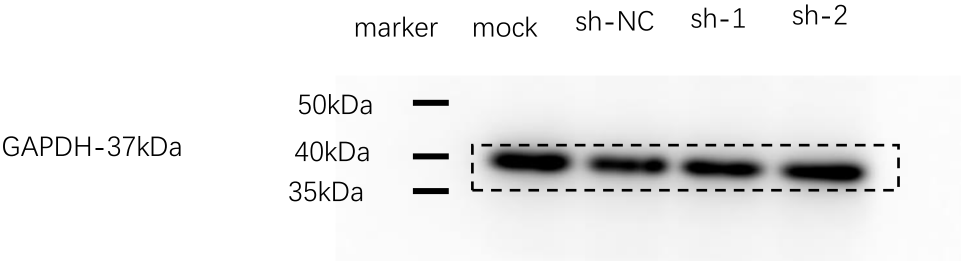

Figure S3E-MDA-MB-231

Vimentin-54kDa

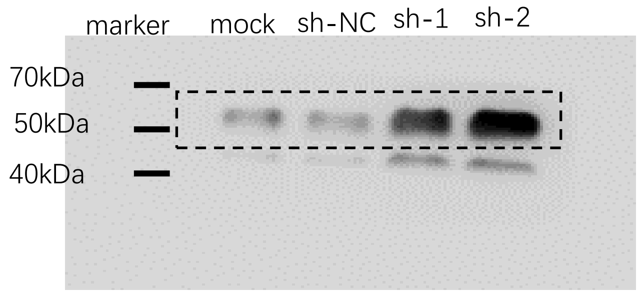

E-cadherin-125kDa

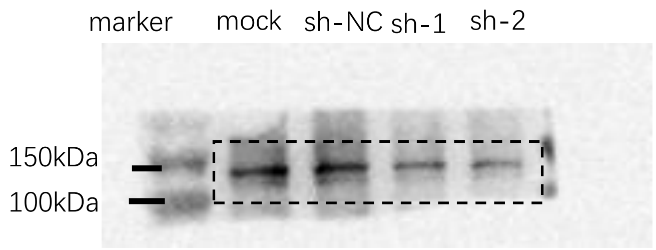

CTNND1-100kDa

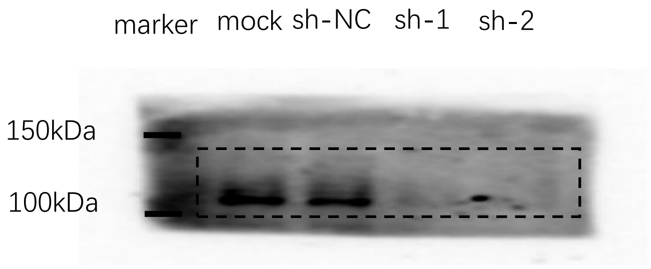

GAPDH-37kDa

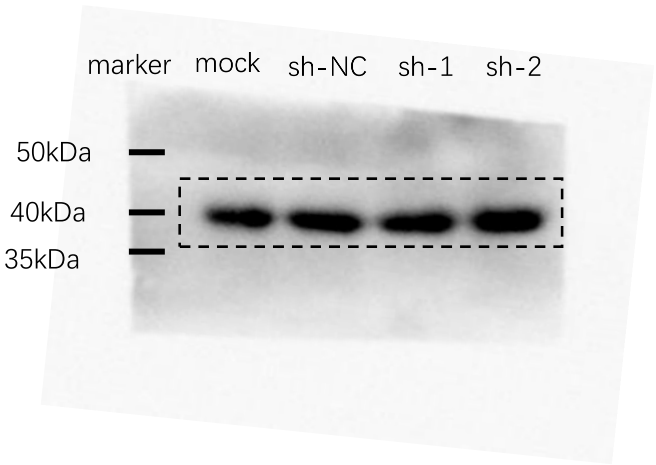

Figure S3E-MDA-MB-468

Vimentin-54kDa

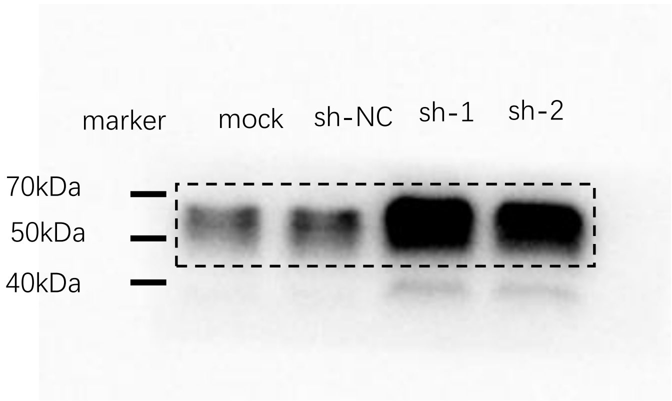

E-cadherin-125kDa

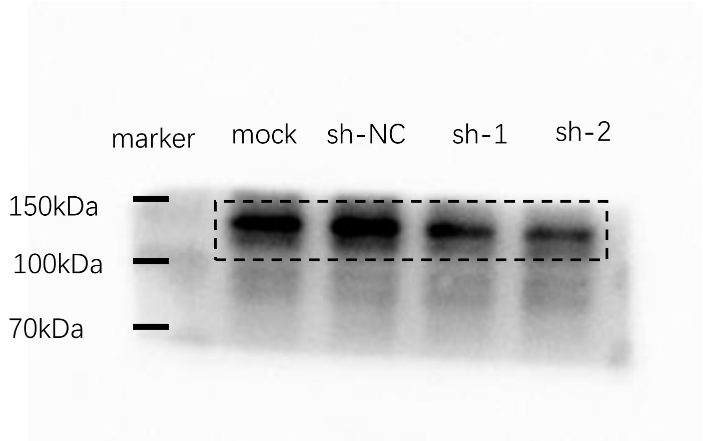

CTNND1-100kDa

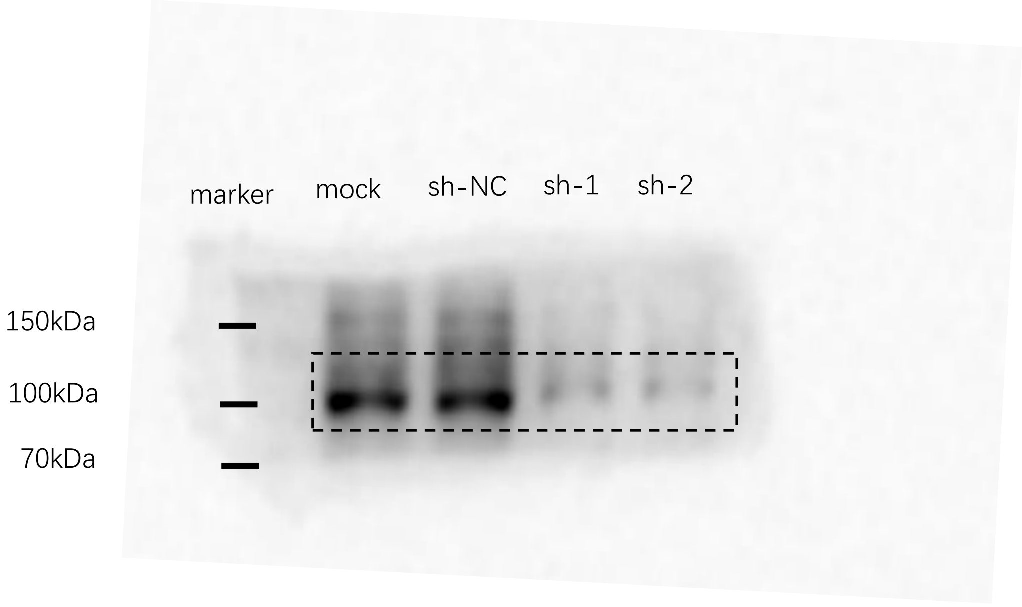

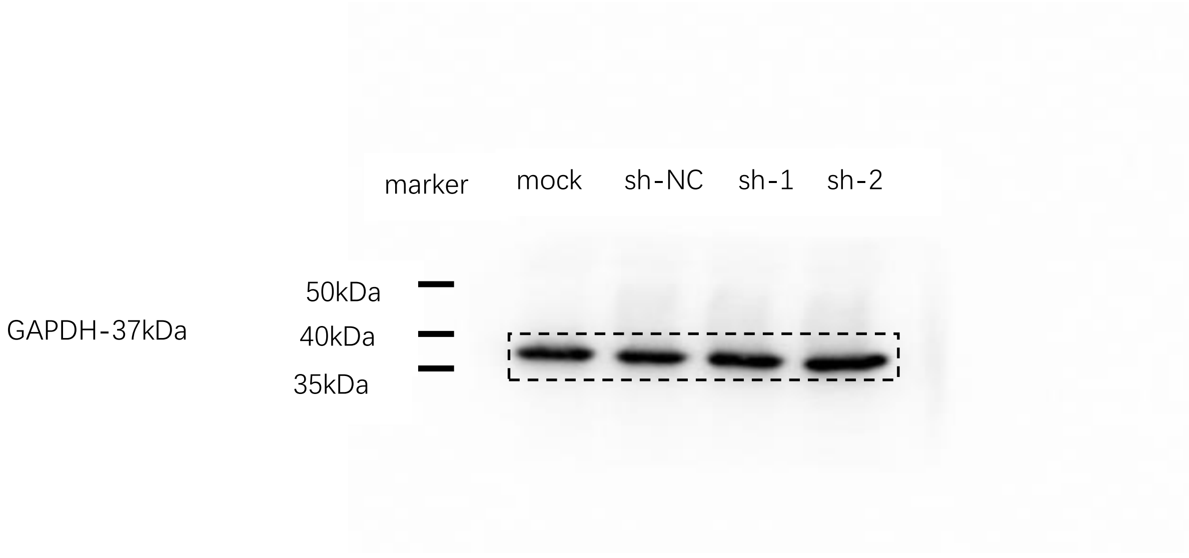

Figure S3E-BT549

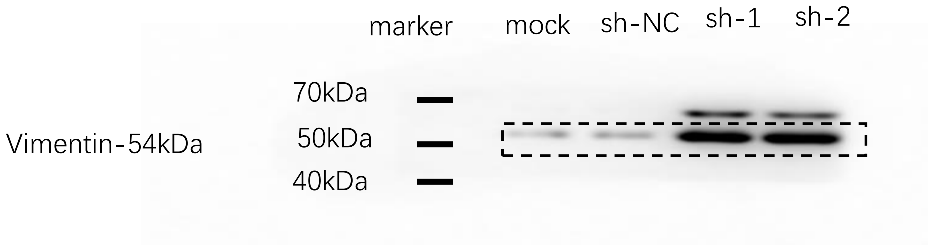

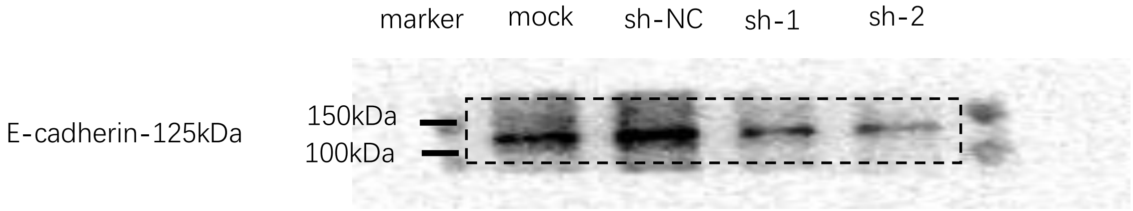

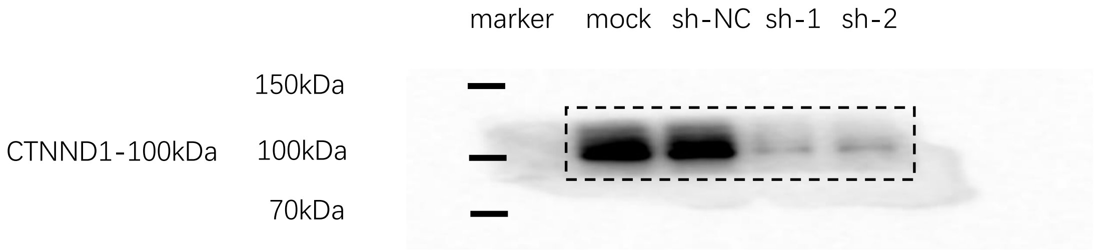

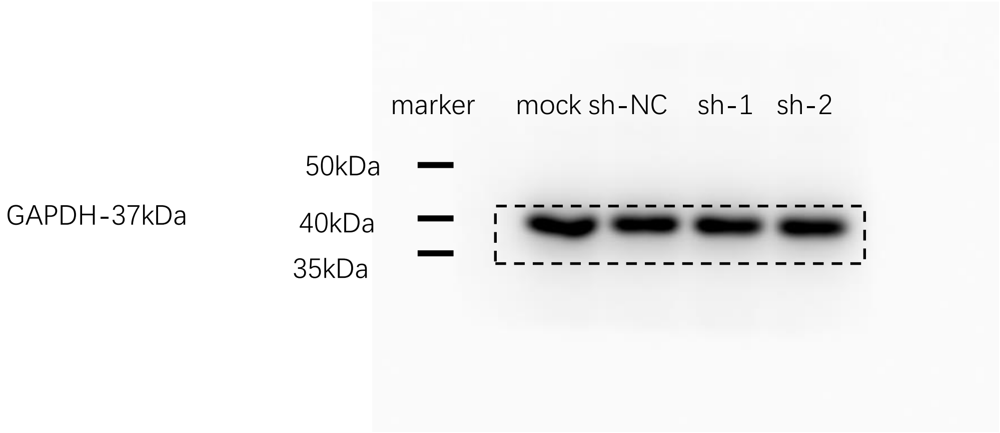

Figure S4B

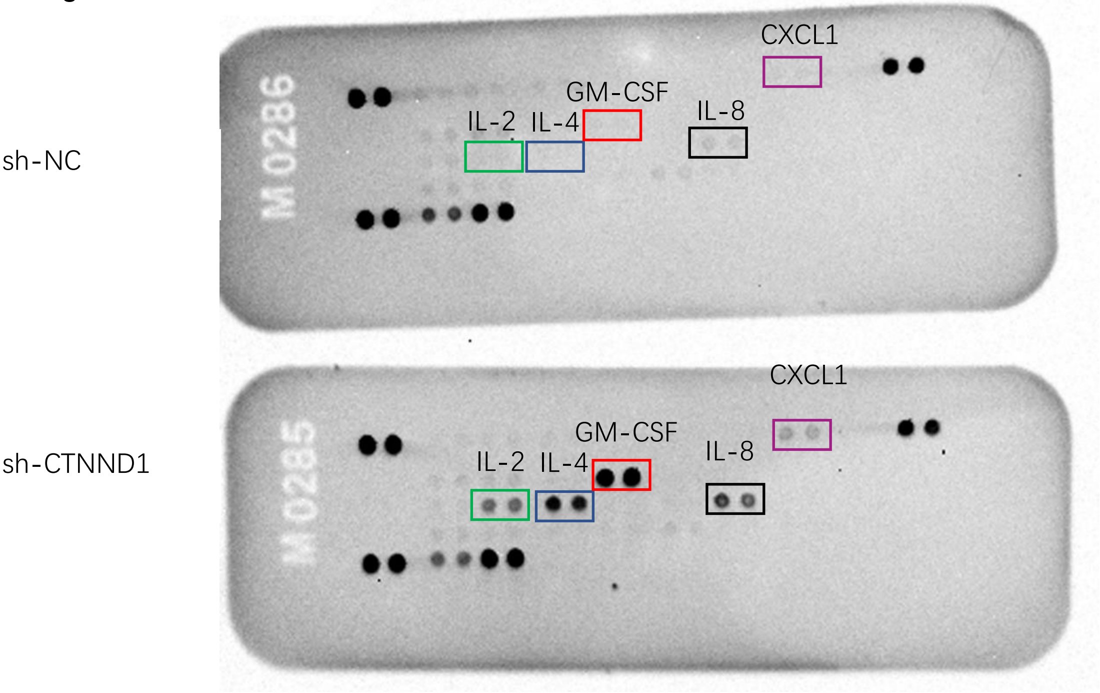

Figure S4C-MDA-MB-231

CXCR4-40kDa

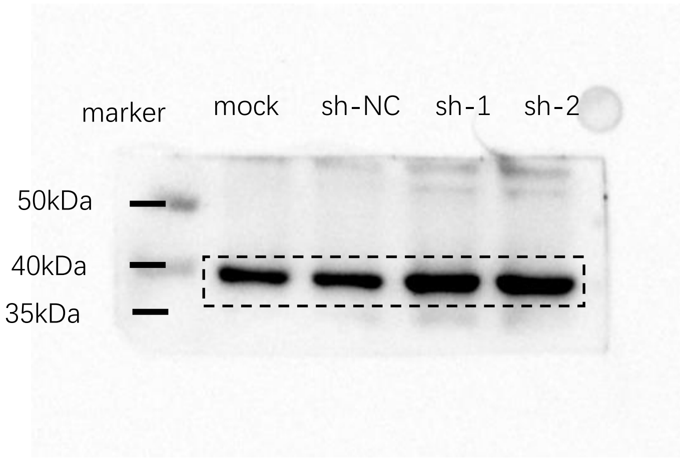

CTNND1-100kDa

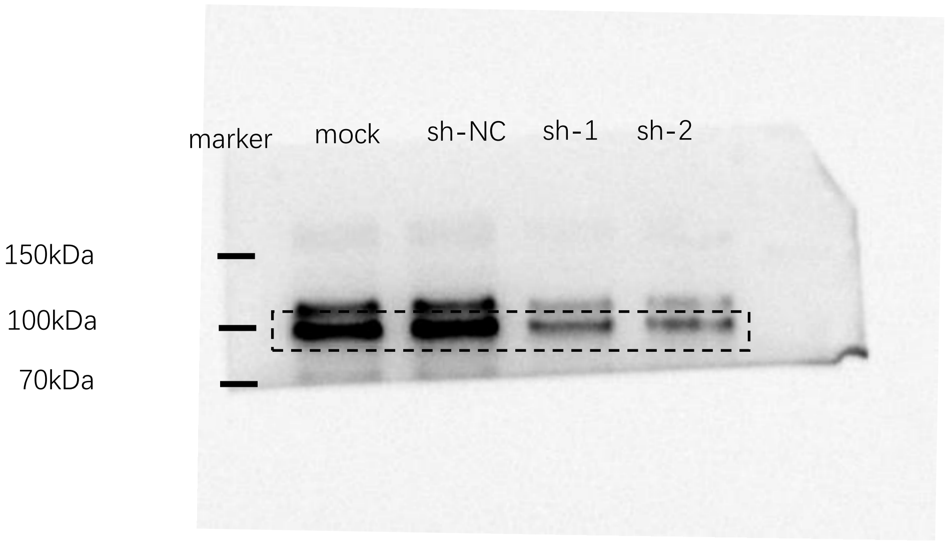

GAPDH-37kDa

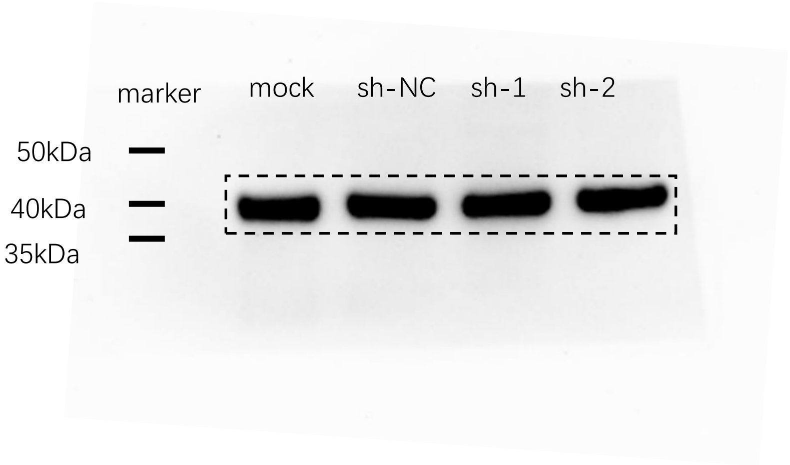

Figure S4C-MDA-MB-468

CXCR4-40kDa

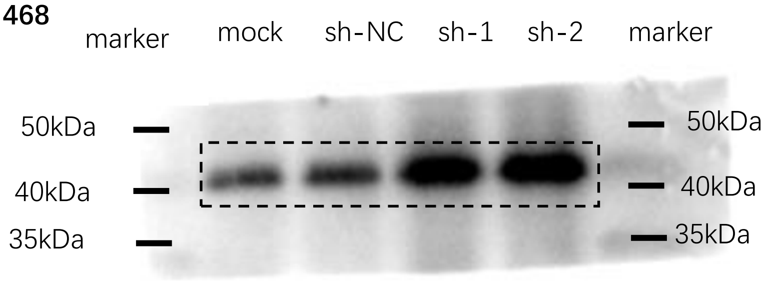

CTNND1-100kDa

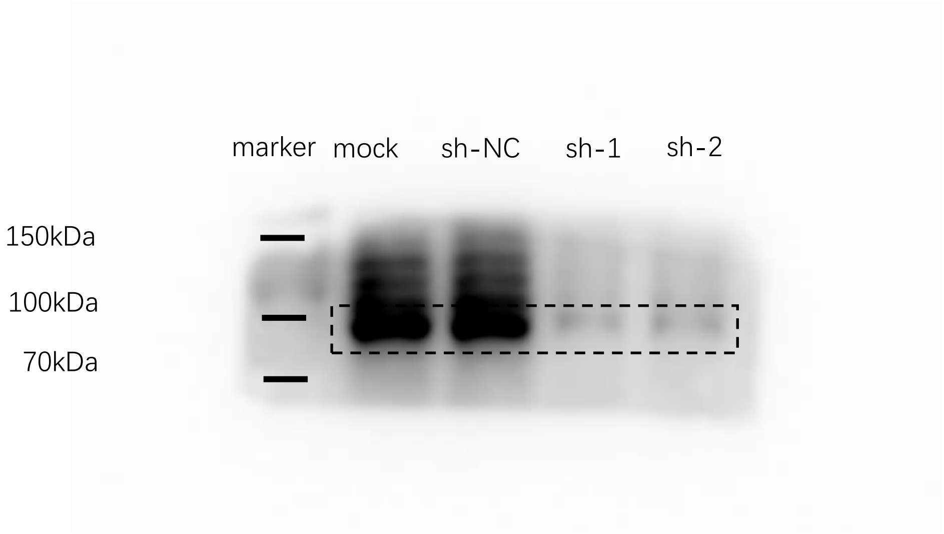

GAPDH-37kDa

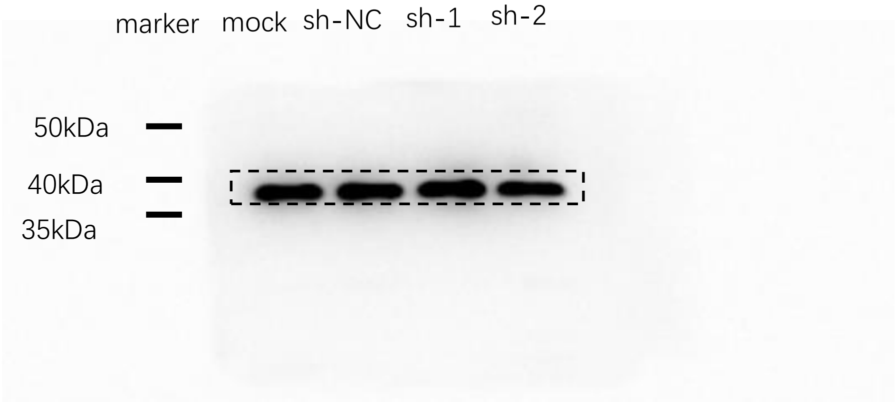

Figure S4C-BT549

CXCR4-40kDa

CTNND1-100kDa

GAPDH-37kDa

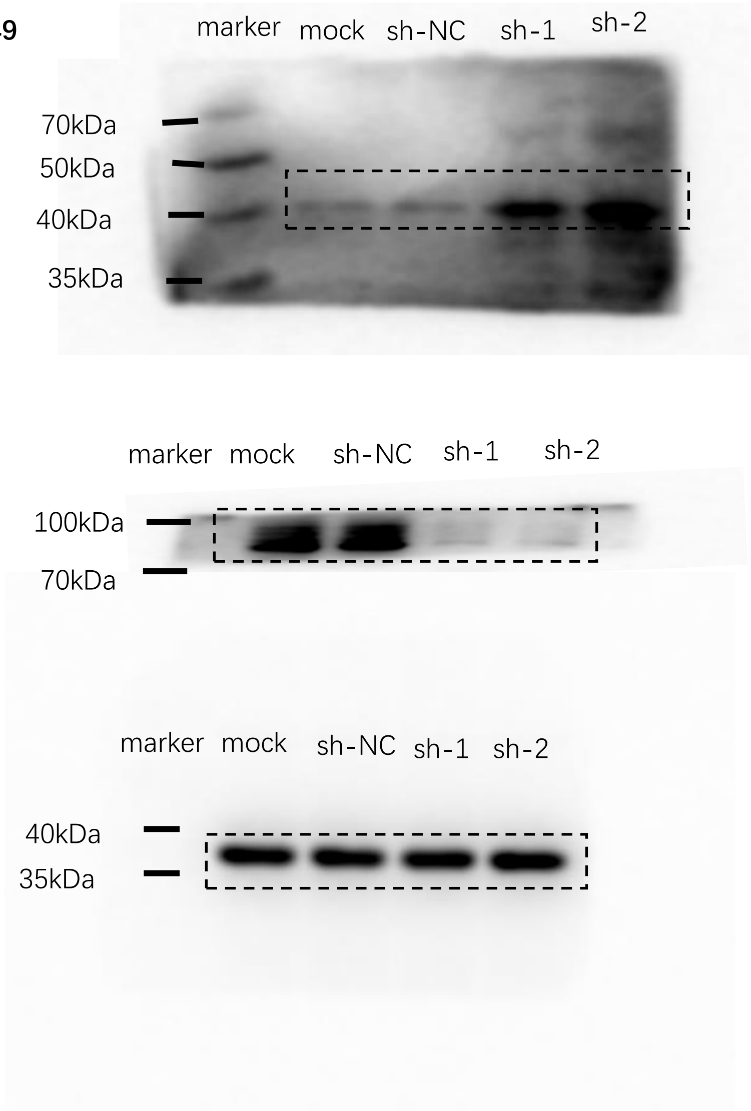

Figure S5B-MDA-MB-231

p-AKT 473-60kDa

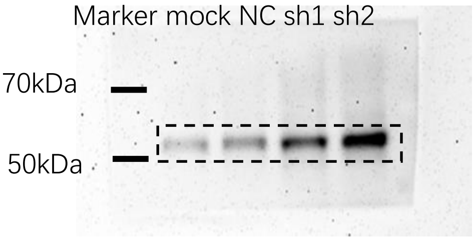

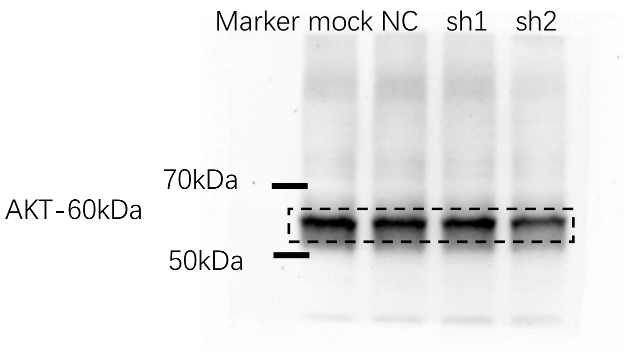

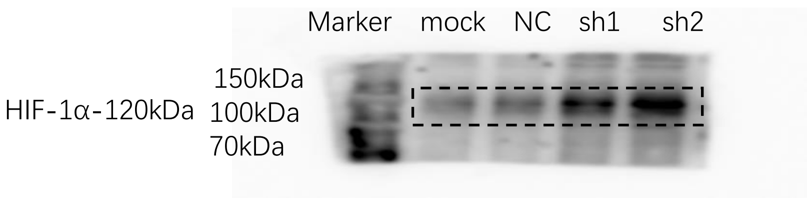

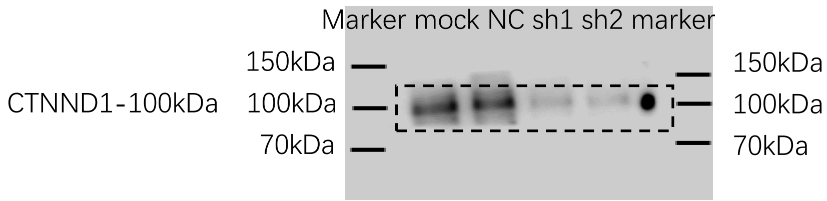

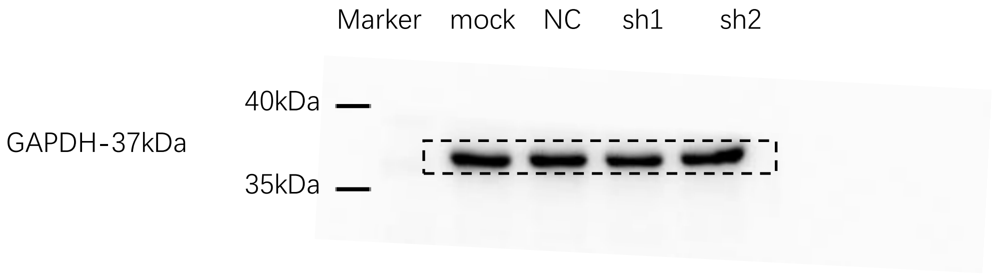

Figure S5B-MDA-MB-468

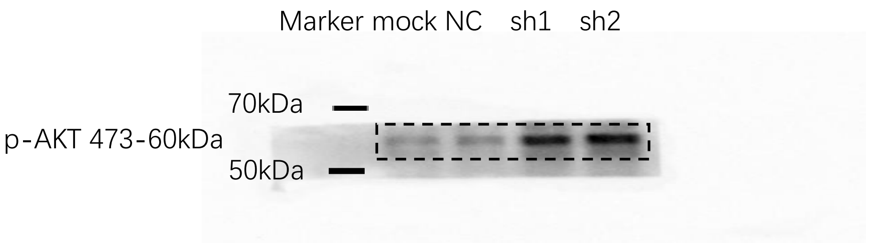

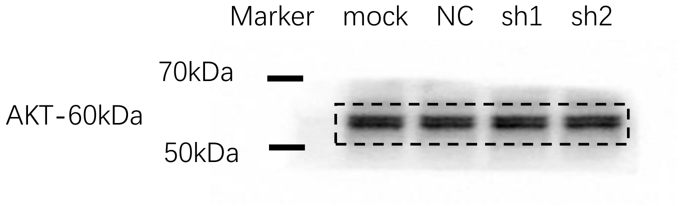

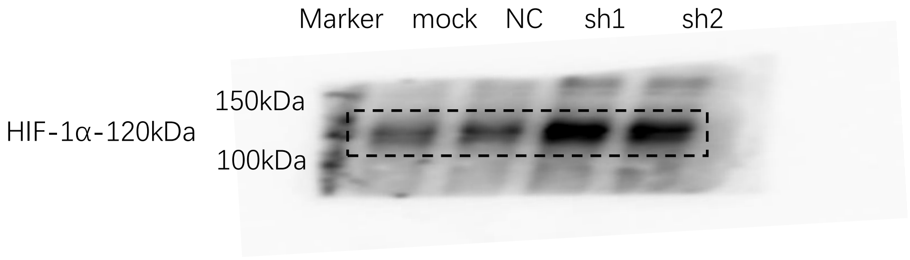

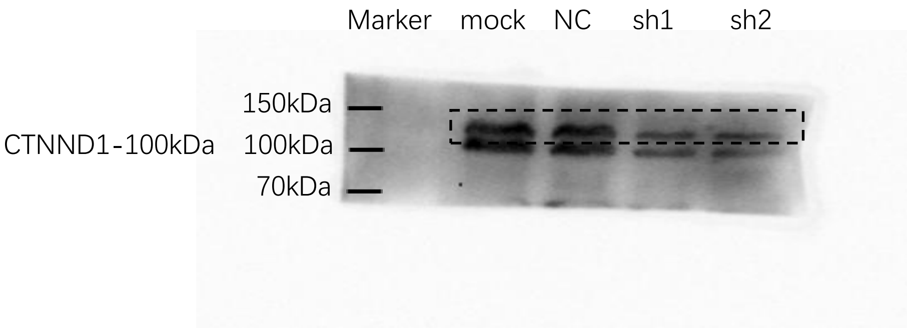

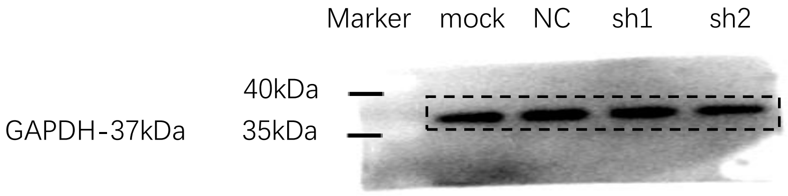

Figure S5C-MDA-MB-231

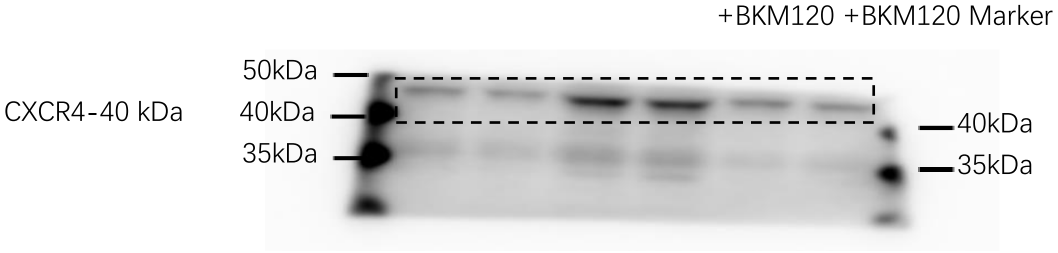

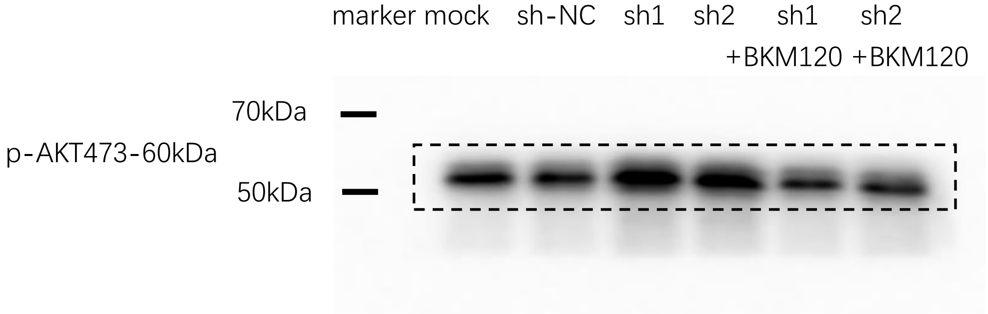

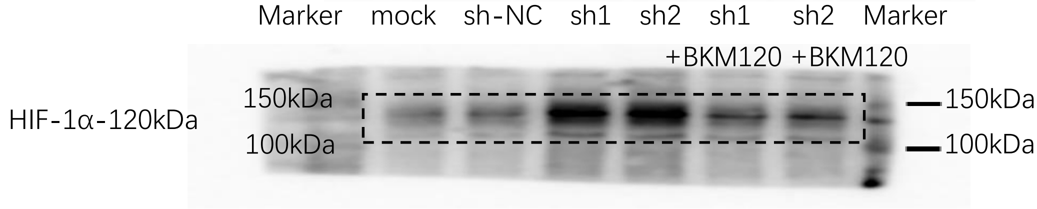

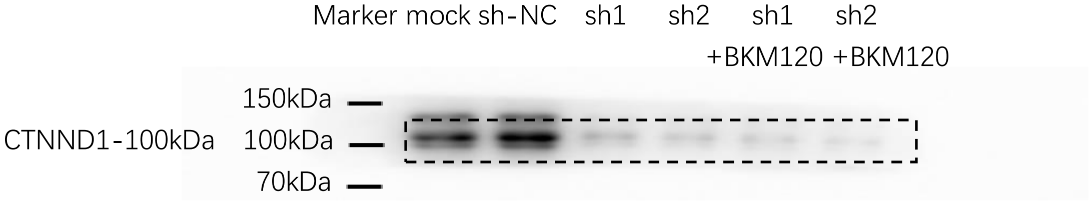

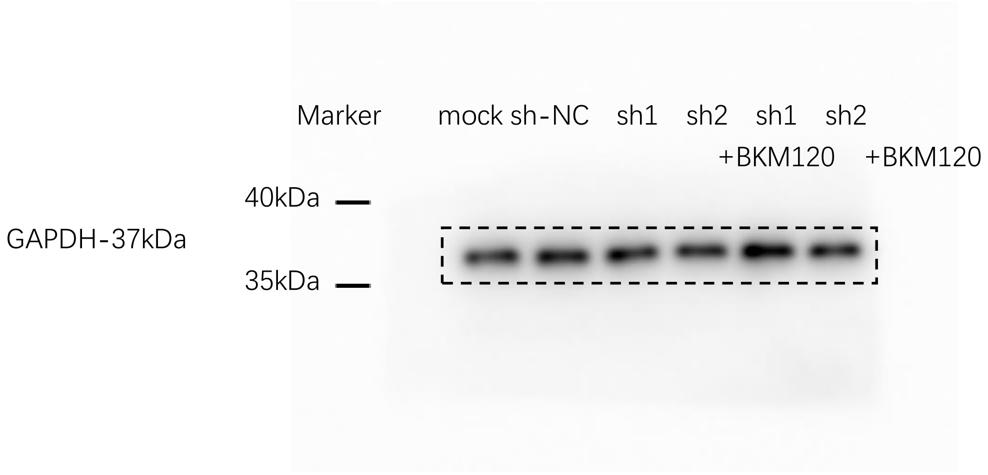

Figure S5C-MDA-MB-468

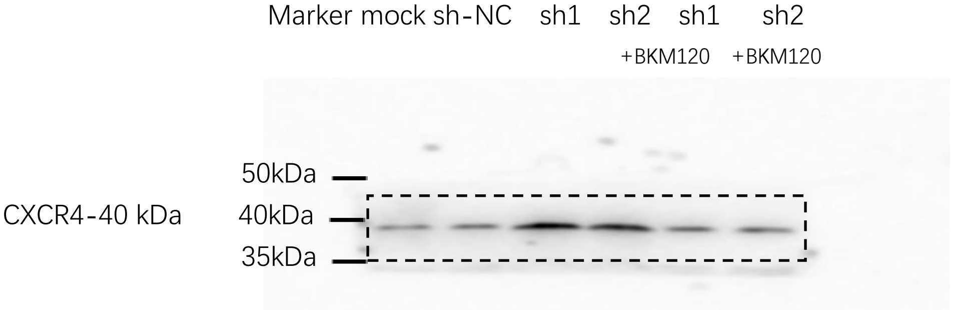

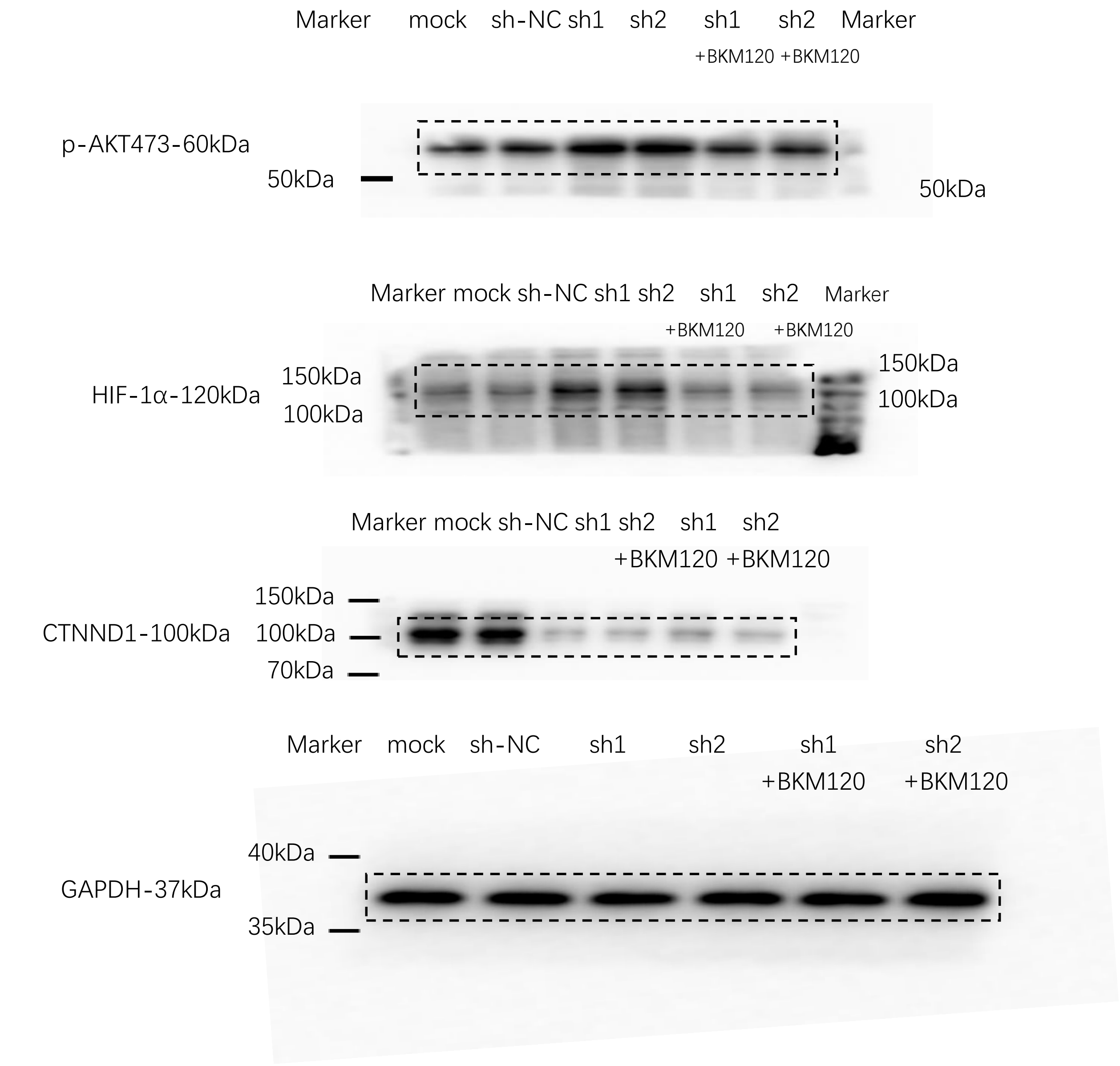
Marker mock sh-NC sh1 sh2 sh1 +BKM120 sh2 +BKM120 Marker
p-AKT473-60kDa
50kDa
50kDa
Marker mock sh-NC sh1 sh2 sh1 +BKM120 sh2 +BKM120 Marker
HIF-1α-120kDa
150kDa
100kDa
150kDa
100kDa
Marker mock sh-NC sh1 sh2 sh1 +BKM120 sh2 +BKM120
CTNND1-100kDa
150kDa
100kDa
70kDa
Marker mock sh-NC sh1 sh2 sh1 +BKM120 sh2 +BKM120
GAPDH-37kDa
40kDa
35kDa

Figure S6B

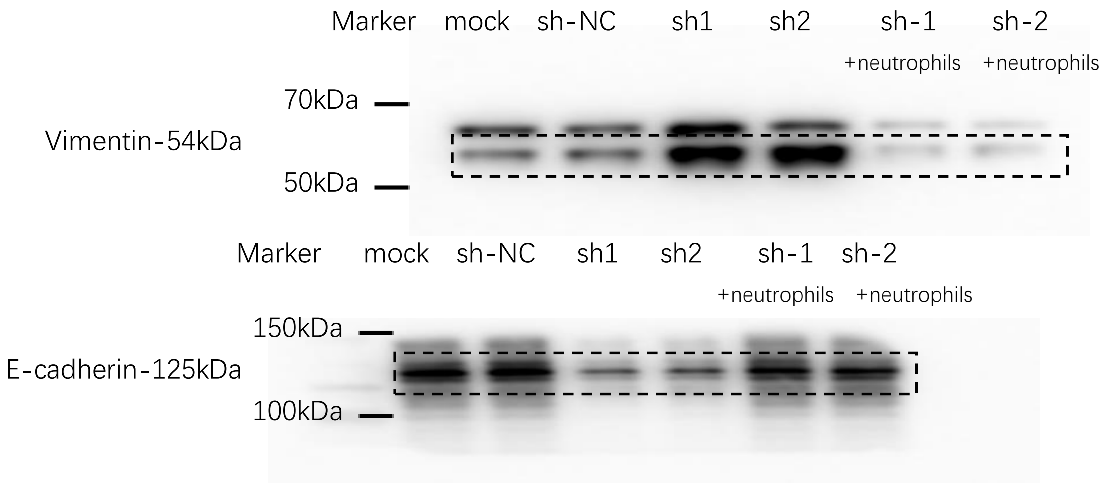
Marker mock sh-NC sh1 sh2 sh-1 +neutrophils sh-2 +neutrophils
Vimentin-54kDa
70kDa
50kDa
Marker mock sh-NC sh1 sh2 sh-1 +neutrophils sh-2 +neutrophils
E-cadherin-125kDa
150kDa
100kDa

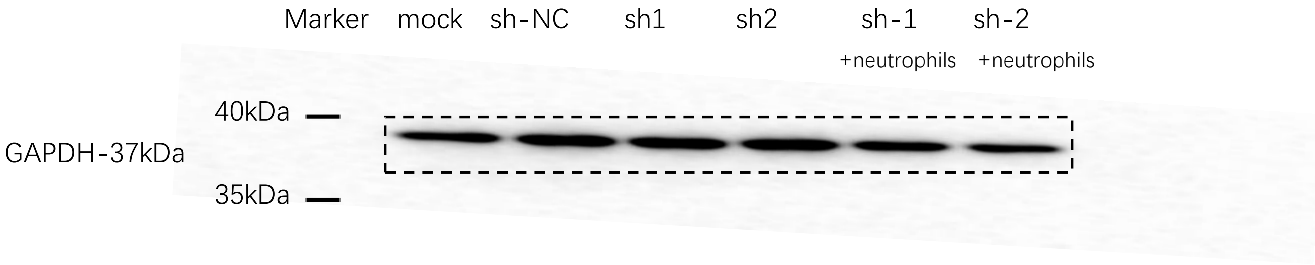

Figure S2C MDA-MB-231

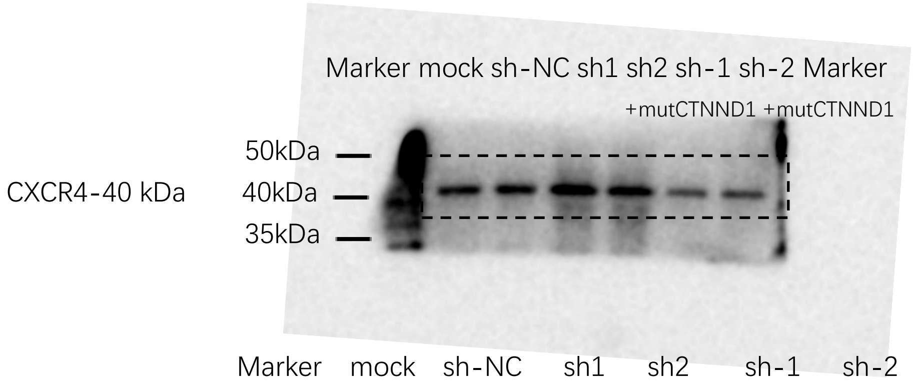

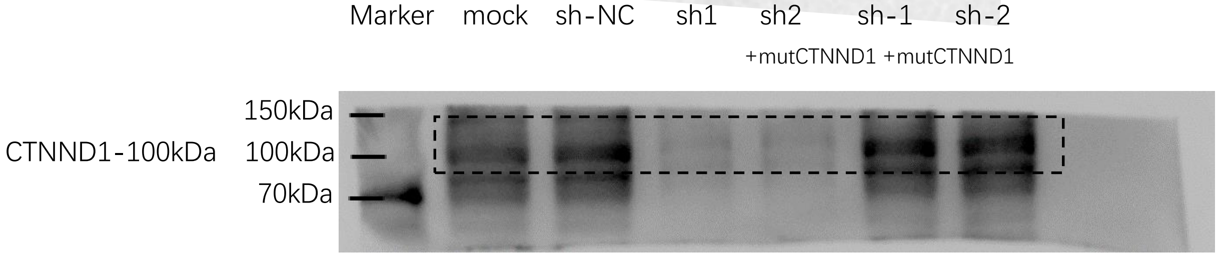

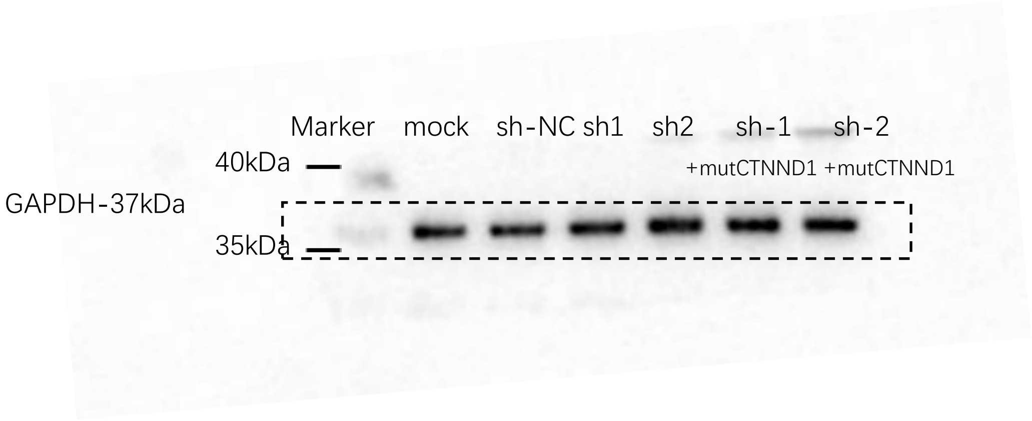

Figure S2C MDA-MB-468

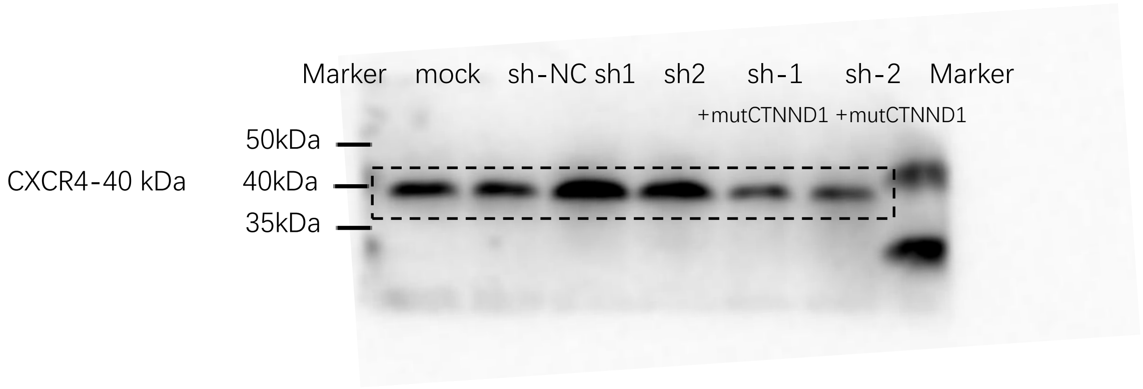

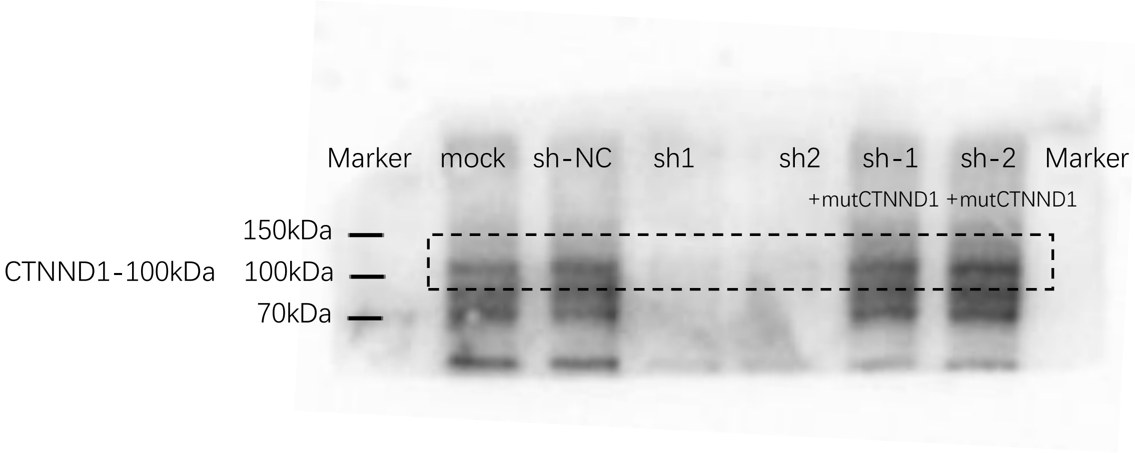

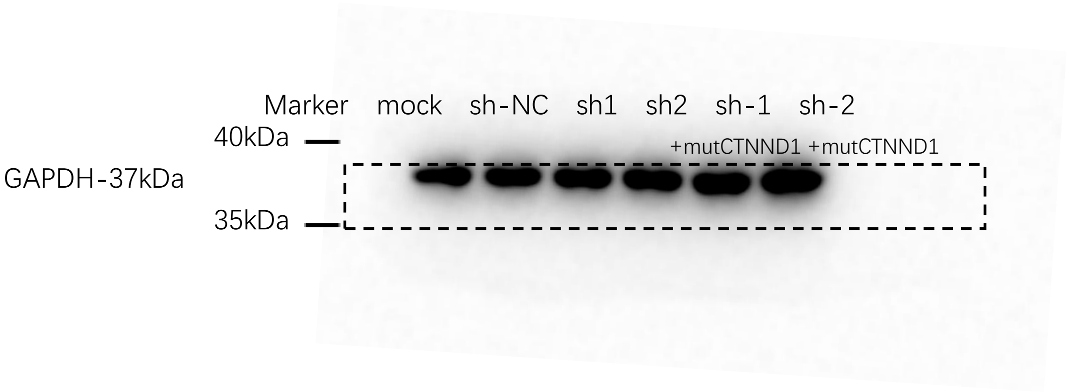

Figure S3B-MDA-MB-231

CXCR4-40kDa

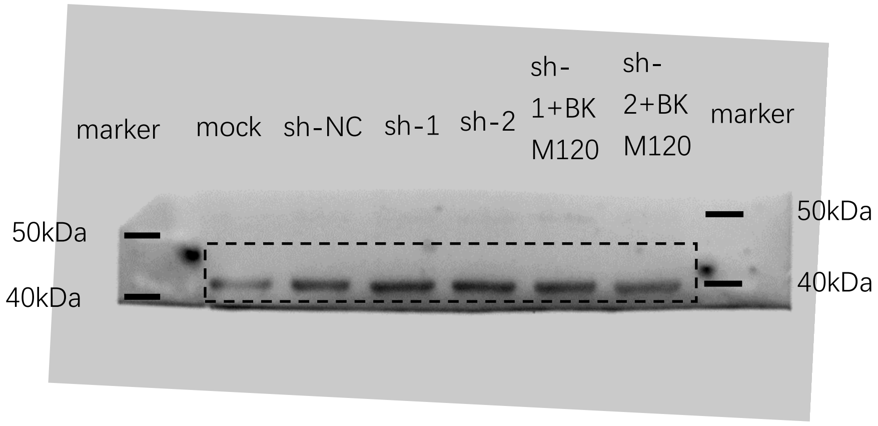

p-AKT 473-60kDa

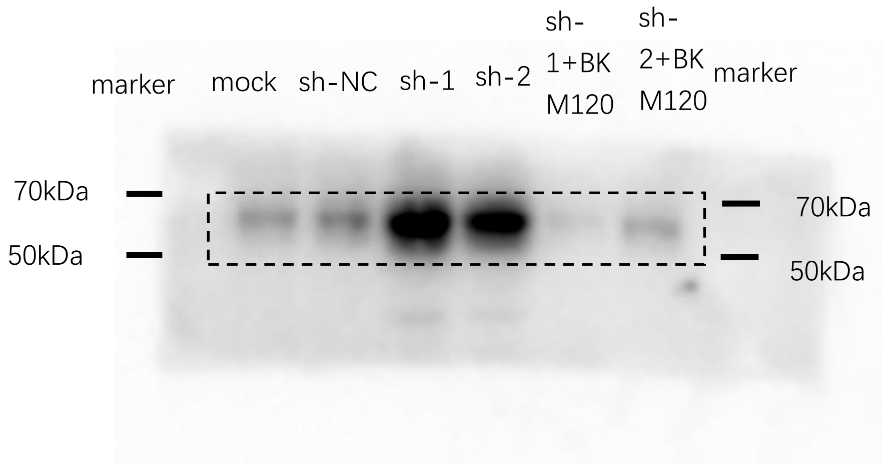

HIF1α-120kDa

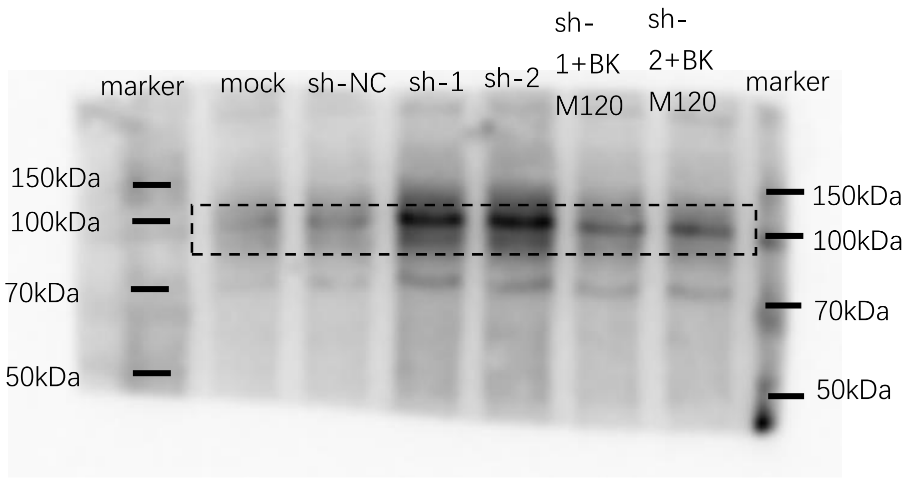

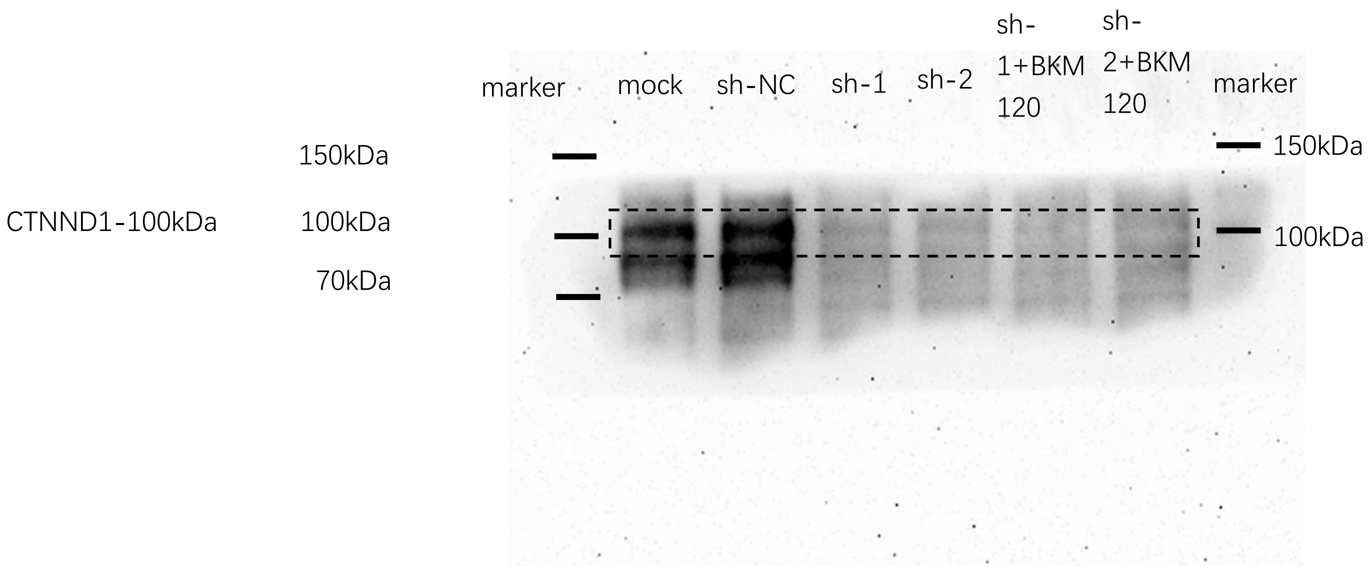

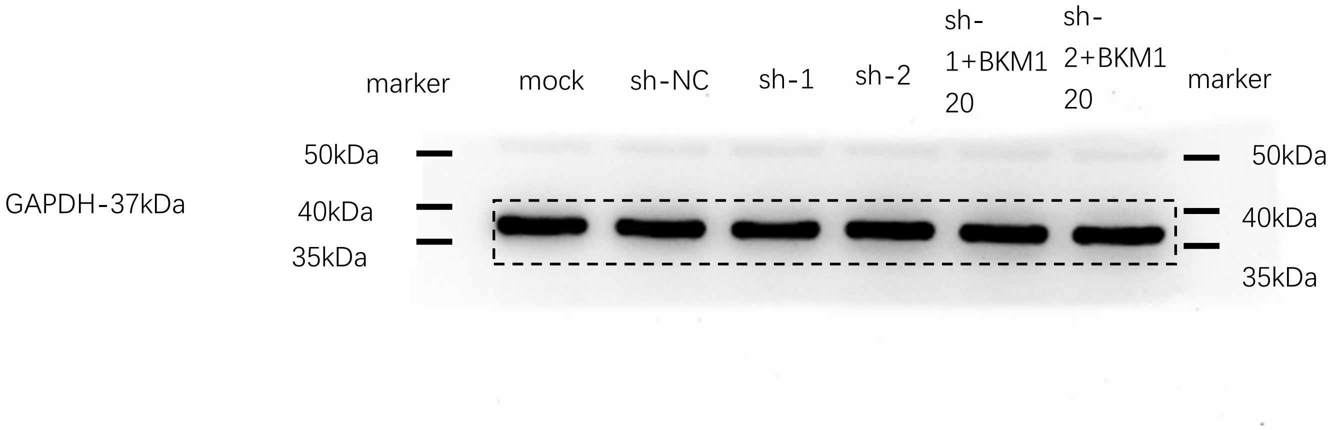

Figure S3D-MDA-MB-468

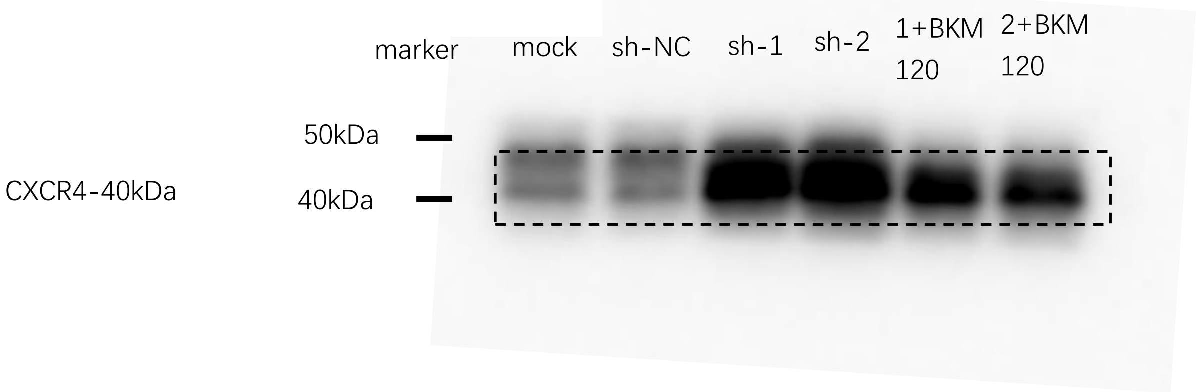

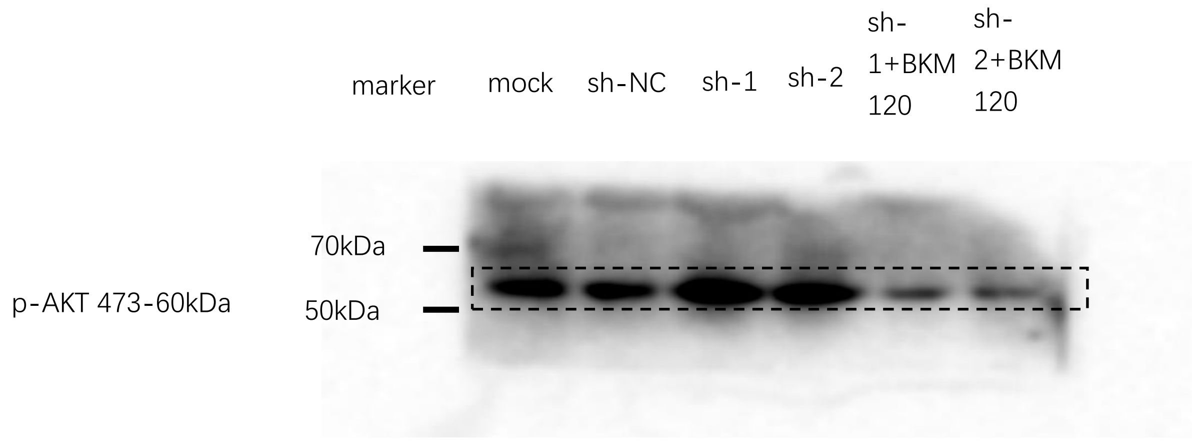
marker
mock
sh-NC
sh-1
sh-2
sh-1+BKM120
sh-2+BKM120
70kDa
p-AKT 473-60kDa
50kDa

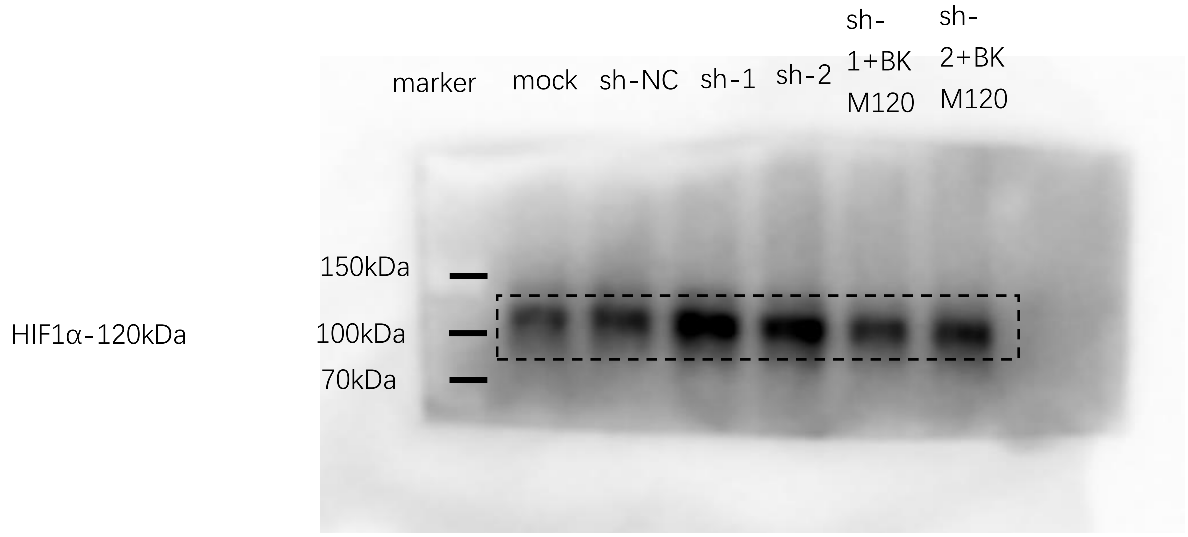
marker
mock
sh-NC
sh-1
sh-2
sh-1+BKM120
sh-2+BKM120
150kDa
HIF1α-120kDa
100kDa
70kDa

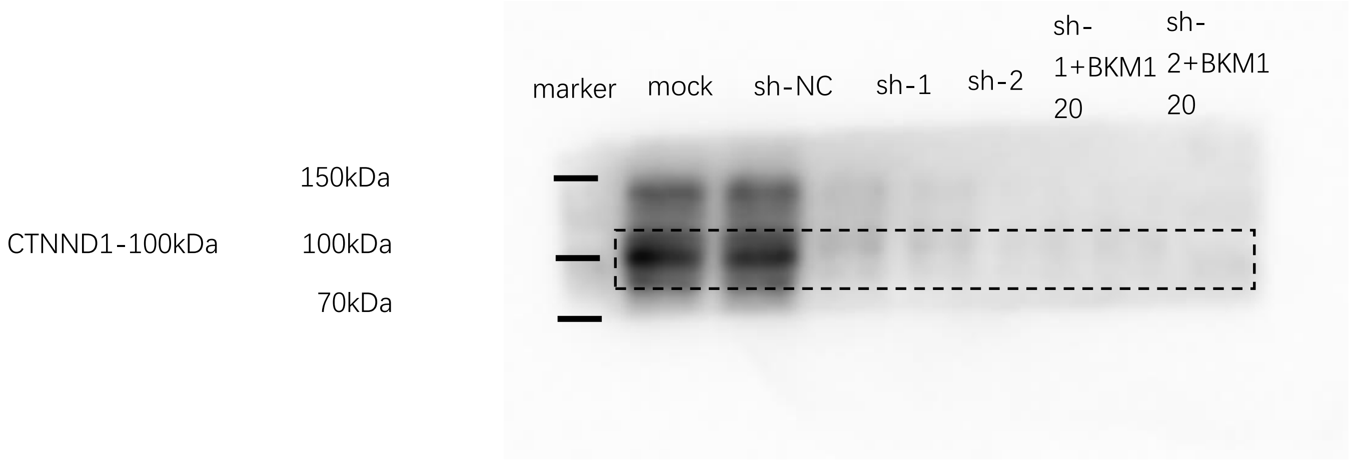
marker
mock
sh-NC
sh-1
sh-2
sh-1+BKM120
sh-2+BKM120
150kDa
CTNND1-100kDa
100kDa
70kDa

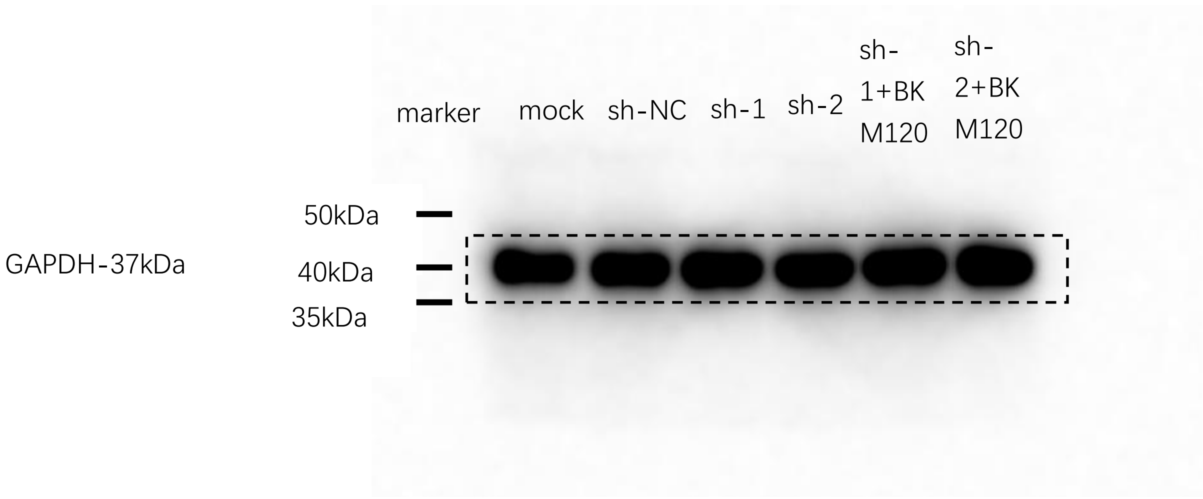
GAPDH-37kDa
marker
mock
sh-NC
sh-1
sh-2
sh-1+BKM120
sh-2+BKM120
50kDa
40kDa
35kDa
